# Correlating Rashba Suppression with Exciton Binding in Low-Dimensional Metal Halide Perovskites

**DOI:** 10.1021/acs.jpclett.6c01964

**Published:** 2026-07-29

**Authors:** Marco Antonio de Andrade Queiroz, Danilo Neves Silveira, Fernando P. Sabino, Carlos M. O. Bastos, Celso R. C. Rêgo, Maurício J. Piotrowski, Alexandre C. Dias, Diego Guedes-Sobrinho

**Affiliations:** † Quantum Chemistry and Thermodynamic Materials Group, Department of Chemistry, Federal University of Paraná, CEP 81531-980 Curitiba, Brazil; ‡ Materials Engineering Department, Engineering School of São Carlos, University of São Paulo, CEP 13566-590 São Carlos, Brazil; ¶ Institute of Physics and International Center for Physics, University of Brasília, Brasília, DF 70919-970, Brazil; § Computational Materials Laboratory, LCCMat, Institute of Physics, University of Brasília, 70910-900 Brasília, Brazil; ∥ 150232 Institute of Nanotechnology Hermann-von-Helmholtz-Platz, Karlsruhe Institute of Technology, 76021 Karlsruhe, Germany; ⊥ Department of Physics, Federal University of Pelotas, Pelotas, RS 96010-900, Brazil

## Abstract

We used DFT-1/2+SOC band structures mapped to Wannier tight-binding Hamiltonians and solved with the Bethe–Salpeter equation to examine how confinement, polymorphism, and metal off-centering tune excitons in Pb- and Sn-iodide perovskites in 3D (MA­(Pb or Sn)­I_3_), quasi-2D (BA_2_(PborSn)­I_4_), and model quasi-1D (BA_3_(PborSn)­I_5_). The 3D polymorphs show a clear trend: as symmetry increases from orthorhombic to tetragonal to pseudocubic, *E*_b_ decreases. At fixed dimensionality, restoring local metal-centered symmetry suppresses Rashba splitting. It reduces or preserves *E*_b_, indicating that SOC affects excitons mainly through band-edge structure rather than direct strengthening of the electron–hole Coulomb kernel. Low-dimensional compounds retain much larger *E*_b_ than 3D analogs, although quasi-1D is not monotonic with quasi-2D. Photoluminescence spectra place the lowest excitons at the optical gap, consistent with bright ground-state excitons.

## Graphical abstract

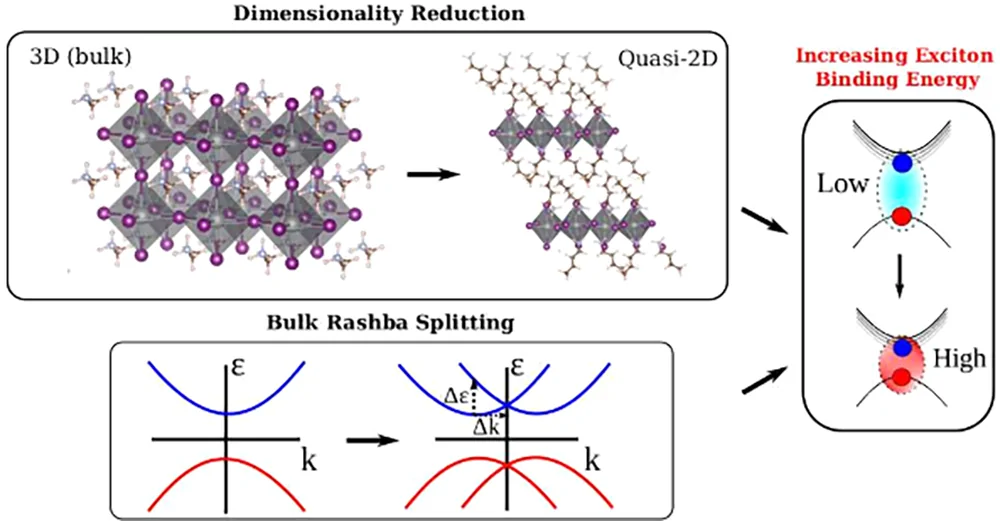

Metal-Halide Perovskites (MHPs) have been identified as promising semiconductors for solar cell applications and other optoelectronic devices, owing to advantages such as a wide compositional range and the ability to form 2D or 3D structures.^1–3^ The atomistic profile becomes quite relevant regarding optoelectronic properties, such as band gaps and optical absorption, as atomic and electronic structures are correlated.^4,5^ Dimensionality reduction in dielectric materials (from 3D → 2D → 1D) increases confinement effects, intensifying the interaction of electron–hole (*e*-*h*) pairs and leading to an enhanced exciton formation.^6,7^ However, available data about these effects, along with the correlations between atomic arrangements and electronic structure, still remain scarce.

3D structures such as MAPbI_3_ (MA^+^ = methylammonium) are arranged by phase, following the orthorhombic → tetragonal → cubic transition as temperature increases.^8,9^ Once the MA^+^ cations are replaced by organic spacer cations (BA^+^ = butylammonium), the structure assumes a quantum-well-like topology as a quasi-2D system.^10^ This affects the physical properties, as low-dimensional systems are known for improved stability and larger band gaps.^11,12^ Hybrid systems such as BA_2_MA_*n*–1_Pb_*n*_I_3*n*+1_ as *n* stacked layers provide a clear illustration on how the dimensionality reduction affects electronic properties, as for *n* = *∞* (bulk) the band gap shows a tendency to reach 1.55–1.67 eV, although for *n* = 1 (quasi-2D) the band gap is increased to 2.43 eV.^12^ Moreover, these correlations still require further investigation once excitonic effects are included.

Lead-based MHPs exhibit strong spin–orbit coupling (SOC) effects, which are associated with local distortions such as metal-off centering within the octahedra, i.e., an induced breaking of inversion symmetry, leading to the bulk Rashba effect.^13–15^ This phenomenon causes spin degeneracy breaking at the band edges, splitting them and consequently altering properties such as band gaps (direct → indirect) and charge-carrier lifetimes.^16,17^ Currently, its impact on the excitonic properties is still unexplored. The exciton binding energies (*E*_b_) have been measured for several MHPs. While MAPbI_3_ displays low *E*_b_ values, ranging from 7 to 130 meV, the monolayered quasi-2D systems BA_2_PbI_4_ and BA_2_SnI_4_ (i.e., for *n* = 1) show 370 and 270 meV, respectively, indicating how structure and composition alter excitonic properties.^18,19^ However, the bulk Rashba effect’s specific role in systematically influencing the exciton binding energies considering MHPs throughout different dimensionalities requires more investigation.

A recent study on how Rashba physics influences nonradiative recombination in the quasi-2D system 3BrMBA_2_PbI_4_ found a 20% increase in carrier lifetime associated with the Rashba splitting.^20^ While their work did not directly consider excitons, their findings suggest that Rashba-active distortions can impact optical and recombination processes. In this study, we address this issue by calculating excitonic properties for Pb- and Sn-based iodide perovskites in three confinement regimes: 3D MAMI_3_, quasi-2D BA_2_MI_4_, and model quasi-1D BA_3_MI_5_, where M is either Pb^2+^ or Sn^2+^. We use the quasi-1D structures as hypothetical models for confinement, not as predicted stable phases. We aim to clarify how dimensionality, polymorphism, and local metal off-centering influence Rashba splitting and exciton binding.

Below, we summarize our methodological approach through the workflow represented in Figure 1 (b).

*i*) First, DFT was employed for the electronic description through the semilocal exchange–correlation functional (based on the Perdew–Burke–Ernzerhof proposal, i.e., PBE) combined with spin–orbit coupling (SOC), in which empirical van der Waals (vdW) based on the Grimme-D3 correction was used.^21–25^ For the total energy and structural optimization, we used the projector-augmented plane wave (PAW) by considering the explicit configurations of the valence electrons, e.g., H (1*s* ^1^), C (2*s* ^2^, 2*p* ^2^), N (2*s* ^2^, 2*p* ^3^), I (5*s* ^2^, 5*p* ^5^), Pb (5*s* ^2^, 5*d* ^10^, 6*s* ^2^, 6*p* ^2^), and Sn (4*d* ^10^, 5*s* ^2^, 5*p* ^2^), within the Vienna *Ab initio* Simulation Package (VASP) code,^26–28^ in which fully relativistic calculations describe the core states. The plane waves were expanded up to a cutoff energy of 480 eV and the Brillouin zone integration was performed considering a *k*-mesh Γ-centered grid of **k** points for 3D structures of 8 × 8 × 8 for pseudocubic, 6 × 6 × 4 for tetragonal, and 6 × 4 × 5 for orthorhombic. For quasi-2D, 6 × 4 × 4 was employed, whereas 6 × 2 × 2 was used for quasi-1D. Finally, the Hellmann–Feynman forces were relaxed until their values were less than 0.010 eV Å^–1^ in each atom.
*ii*) Moving for the band gap calculations, alternatively to the hybrid functionals and GW approximation (given their high computational cost associated),^2^ we have applied the widely used DFT-1/2+SOC as a low cost approach.^4–6,12,29,30^ Based on the Slater transition technique, the valence band is half-occupied by the 5p states of I, and the conduction band is dominated by the 6p states of Pb or the 5p states of Sn. Here, a modified Kohn–Sham potential is built up via $V_{\mathrm{mod,KS}}=V_{\mathrm{KS}}\left(\mathrm{r⃗}\right)-V_{s}\left(\mathrm{r⃗}\right)$ , where $V_{\mathrm{KS}}\left(\mathrm{r⃗}\right)$ represents the standard Kohn–Sham potential and $V_{s}\left(\mathrm{r⃗}\right)$ is the self-energy potential, which is calculated through $V_{s}\left(\mathrm{r⃗}\right)=\Theta \left(\mathrm{r⃗},CUT\right)\left[V_{ae}\left(\mathrm{r⃗}\right)-V_{ae-1/2}\left(\mathrm{r⃗}\right)\right]$ from the optimized CUT parameter which maximize the band gap value (Figure S1 contains all CUT optimizations).
*iii*) Through the maximally localized Wannier functions we have built up the Hamiltonians—from the Wannier90 code^31^—for all the systems by aiming to access the excitonic properties through a tight-binding (TB) approach, in which the Bethe–Salpeter Equation (BSE)^32^ is solved and the ground state of the exciton is obtained from a low computational effort in detriment to the *ab initio* approaches.
*iv*) Finally, TB-BSE solutions were obtained through the WanTiBEXOS code by taking a 3D Coulomb potential for the *e*-*h* pairwise interaction,^33^ from which the ground state of the exciton (i.e., its optical band gap, *E*_opt_), exciton band structure, and the optical absorption coefficient were calculated. Thus, the exciton binding energies were calculated through the difference between the fundamental and optical band gap, i.e., *E*_b_ = *E*_g_ – *E*_opt_. Furthermore, the linear optical response was calculated within both the independent-particle approximation (IPA) and BSE to capture excitonic quasi-particle effects, as well as the photoluminescence peaks at 300 K.^34^ Details regarding possible configurations and other code definitions are presented in the Supporting Information (Table S1). Although the systems are referred to as quasi-2D and quasi-1D based on the connectivity of the inorganic framework, the octahedral layers/wires are separated only by the organic spacer cations, without vacuum interfaces. Therefore, the simulations employ fully periodic three-dimensional crystal structures, in which a 3D Coulomb interaction was used throughout the calculations.

**Figure 1:**
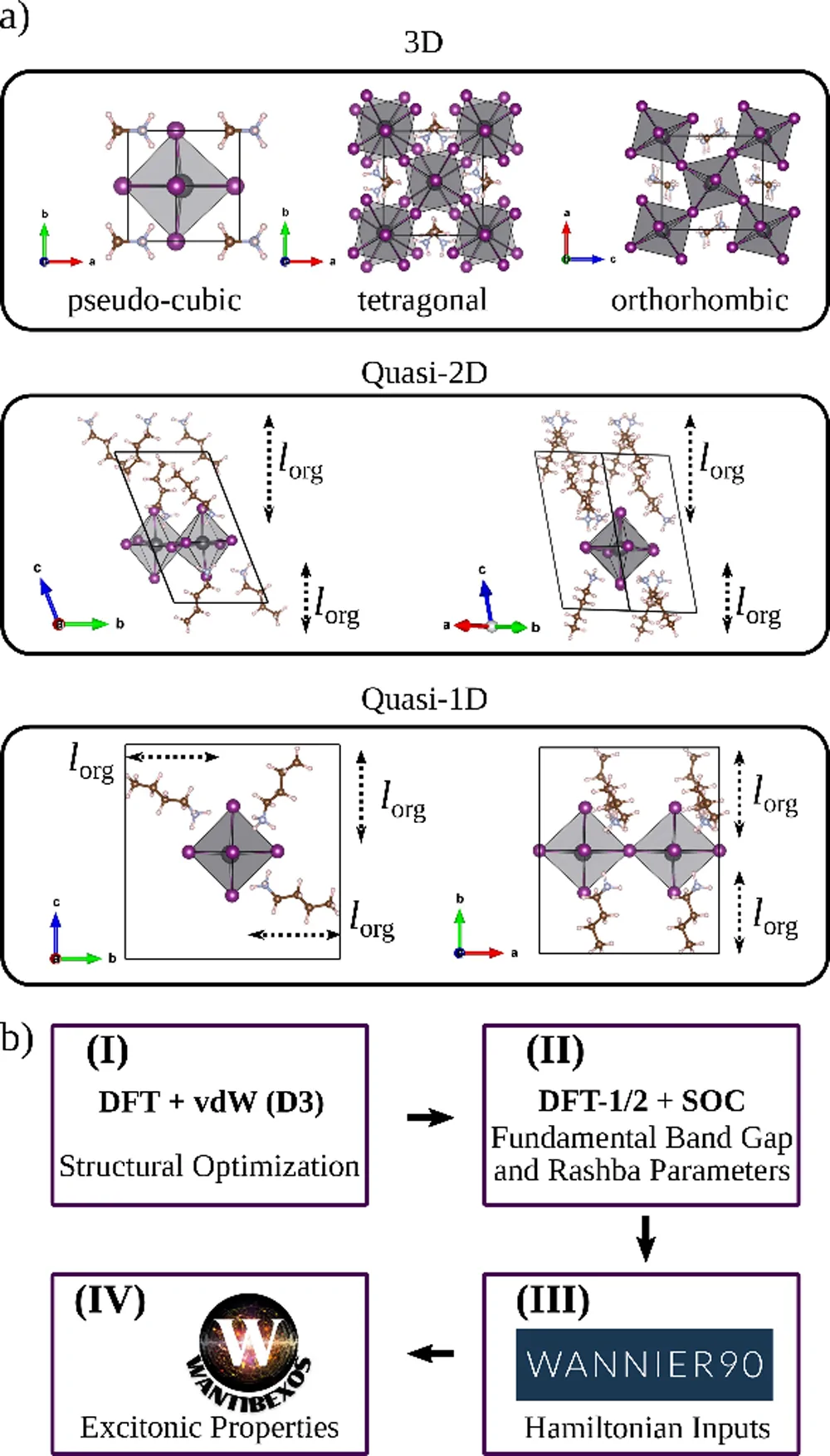
a) Unit cell representation of the investigated structures, from 3D (pseudocubic, tetragonal, and orthorhombic phases) to quasi-2D and quasi-1D (*l*_org_ depicts the length of the organic layer through the BA^+^ spacer cation). b) Methodology workflow: (I) geometry optimization done through DFT with the addition of vdW correction; (II) electronic structure characterization (including the fundamental band gap and Rashba parameters determination) done with DFT through the employment of DFT-1/2 correction and addition of SOC effects; (III) Wannierization step to generate the Hamiltonian inputs; (IV) TB-BSE, done through WanTiBEXOS, to calculate the excitonic properties.

In a comparative analysis of our structural data with experiments (as detailed in Table S2 in the Supporting Information),^11,35–37^ we have obtained small deviations for the lattice parameters through the optimization process of the 3D and quasi-2D, which indicates that the DFT+D3 protocol is suitable for the different dimensionalities. For instance, for *a*, *b*, and *c* parameters in MAPbI_3_ (MASnI_3_) as pseudocubic, tetragonal, and orthorhombic, the deviations did not overcome 2.5% (2.8%). For quasi-2D monolayered BA_2_PbI_4_ (BA_2_SnI_4_) systems, the deviations were less than −1.0% (−1.2%). Furthermore, the larger deviation observed along the organic stacking direction, as depicted in Figure 1(a) as *l*_org_, is consistent with previous DFT studies and reflects the strong temperature dependence of the organic spacer arrangement.^12,19,38^ Conversely, for quasi-1D structures, it is important to note that they should be regarded as hypothetical model systems intended to investigate confinement trends, not as predicted stable phases; therefore, there are no experimental data for comparative analysis.

On the other hand, for all the dimensionalities, the metal off-centering magnitude (as a symmetry-breaking pattern associated with the bulk Rashba effect over strong metal spin–orbit coupling) is characterized by the local octahedral distortions from M–I bond lengths. These bonds split in short–large lengths, given the MA^+^ cations in the 3D systems employ different distortion patterns into the pseudocubic, tetragonal, and orthorhombic structures. While this M–I bond splitting is pronounced for pseudocubic and tetragonal structures, this feature vanishes entirely for the orthorhombic structures, given the alignment of the tilting between different octahedral layers, yielding simultaneously nearly centrosymmetric octahedra and equally aligned MA^+^ cations with canceled dipole moments. With platonic-like octahedra (as shown in Table S2), the orthorhombic phase does not present bond-length asymmetries responsible for local inversion-symmetry breaking.

To distinguish the effects of local inversion-symmetry breaking from those of dimensional confinement, we made two structural variants for each composition and dimensionality. The fully relaxed structures are called metal-off because they retain the local M–I bond-length asymmetry and octahedral distortions associated with the local distortion known as the metal off-centering process. We created the metal-on structures by moving the metal atom to the center of the octahedron (metal on-centering process), removing the short and long M–I bond difference, and setting the relevant M–I–M angles to 180°, while keeping the relaxed unit-cell volume the same. These metal-on structures serve as idealized high-local-symmetry references, not as new thermodynamic phases. This approach lets us test how restoring local symmetry affects Rashba splitting and exciton binding at fixed composition and dimensionality. The octahedral-tilt patterns are shown using Glazer notation in Figure S2 in the Supporting Information.^44^

The values of electronic band gap energies (*E*_g_) for both Pb and Sn systems as 3D systems in pseudocubic and tetragonal, monolayered quasi-2D (i.e., *n* = 1), and model quasi-1D fully relaxed are summarized in Figure 2. Our DFT-1/2+SOC protocol provided a set of *E*_g_ values in excellent agreement with experiments (*E*_g,exp_). For instance, MAPbI_3_ as pseudocubic (tetragonal) reaches 1.52 eV (1.54 eV), deviating in 2.7% (1.3%) given the 1.48 eV^40^ (1.52 eV)^39^ experimentally reported. Similarly to MASnI_3_ as pseudocubic (tetragonal), *E*_g_ = 1.13 eV (1.25 eV) value deviates only −15% (3.3%) from the 1.33 eV^1^ (1.21 eV),^11^ so that this behavior is kept for the orthorhombic MAPbI_3_ with deviation not higher than −5.9% (as shown in Table S3 in the Supporting Information, which contains all the obtained *E*_g_ and experimental references when available).

**Figure 2:**
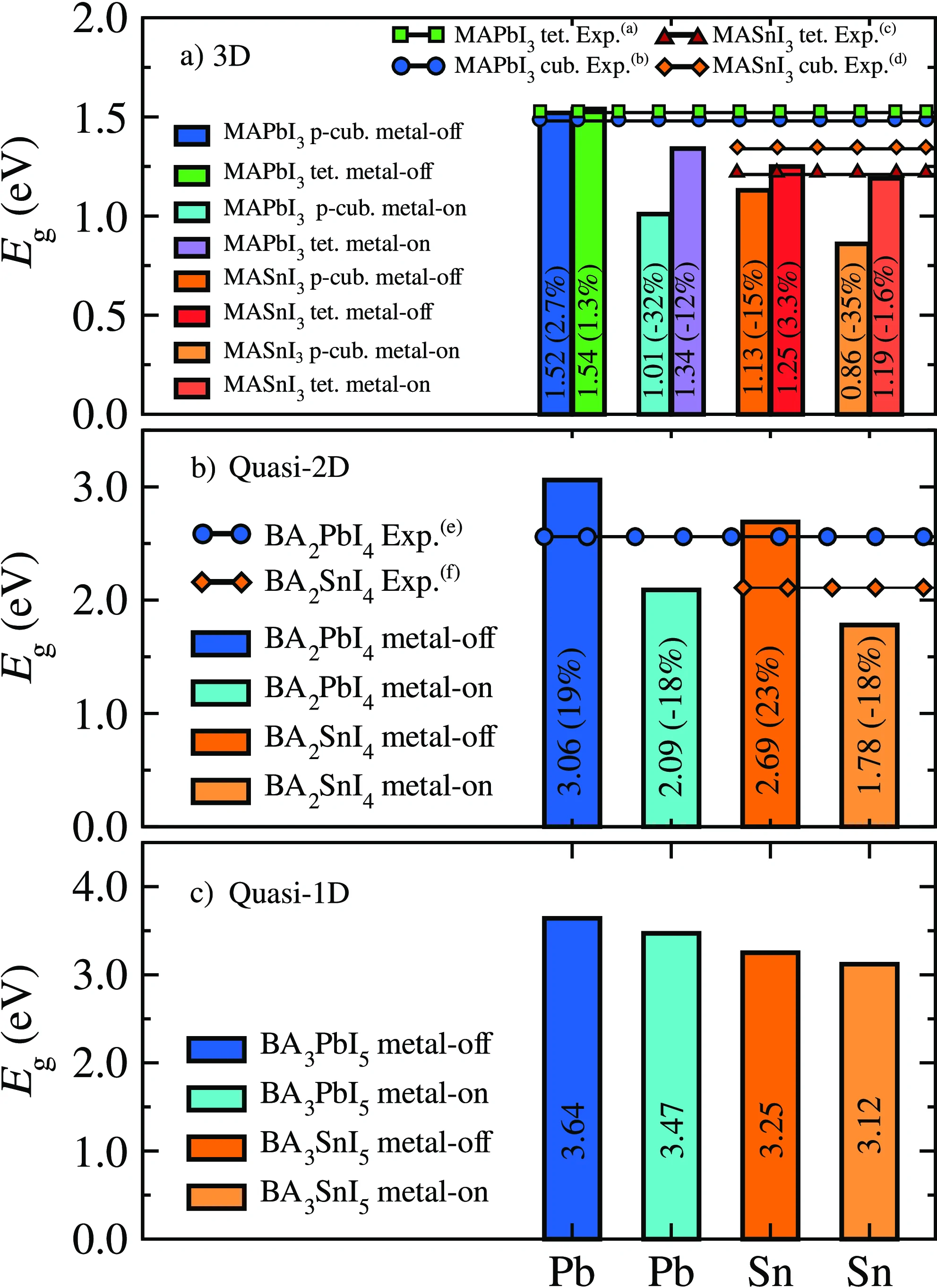
Electronic band gap energies based on DFT-1/2+SOC protocol for the a) 3D MAPbI_3_ and MASnI_3_ structures as metal-off in pseudocubic and tetragonal phases, b) quasi-2D BA_2_PbI_4_ and BA_2_SnI_4_, and c) quasi-1D BA_3_PbI_5_ and BA_3_SnI_5_, all the configurations with their metal-on counterparts. The horizontal lines refer to experimental values for MAPbI_3_ in tetragonal phase (green squares, 1.52 eV),^39^ MAPbI_3_ in pseudocubic phase (blue circles, 1.48 eV),^40^ MASnI_3_ in tetragonal phase (red triangles, 1.21 eV),^11^ MASnI_3_ in pseudocubic phase (orange diamonds, 1.33 eV),^41^ BA_2_PbI_4_ (2.56 eV),^42^ and BA_2_SnI_4_ (2.18 eV).^43^

For 3D → quasi-2D changing as an enhancement of the confinement effects, BA_2_PbI_4_ (BA_2_SnI_4_) reaches a fundamental band gap of *E*_g_ = 3.06 eV (2.69 eV). These values are higher by 19% (23%) than the optical band gap of *E*_opt_ = 2.56 eV (2.18 eV) from experiments based on temperature-dependent photoluminescence and transient absorption (TA) spectroscopy (electroabsorption (EA) spectroscopy) techniques in which the excitonic effects are captured (so that *E*_opt_ concerns the exciton ground state). The values for the hypothetical quasi-1D systems BA_3_PbI_5_ and BA_3_SnI_5_ are *E*_*g*_ = 3.64 and 3.25 eV, respectively, showing the increase of the confinement effect associated with the enhancement of the opening band gap. These results provide a quantitative picture of the dimensionality reduction correlated with the fundamental band gap broadening, given the reduction of electronic states on the band edge.

One observes in Figure 2 a decrease in the band gap—i.e, *E*_g_(off) > *E*_g_(on)—for the Pb and Sn systems in all dimensionalities. The metal off-centering → on-centering changes yield a higher *sp* hybridization that forms the valence top (which also lies closer to the vacuum) when equatorial and apical M–I–M angles are 180° for the platonic-like configurations (i.e., metal on-centering and no octahedral tilting). For instance, the *a* ^0^ *a* ^0^ *a* ^0^ platonic centrosymmetric as 3D pseudocubic (tetragonal) configuration yields gap energies of 1.01 eV (1.34 eV) for the MAPbI_3_, whereas for MASnI_3_ the *E*_g_ was reduced to 0.86 eV (1.19 eV).

For the quasi-2D systems this behavior is more pronounced given the reduced BA_2_PbI_4_ (BA_2_SnI_4_) *E*_g_(off → on) values of 3.06 eV → 2.09 eV (2.69 eV → 1.78 eV). We observed that quasi-2D MHPs are more sensitive to local distortions and octahedral geometry than their bulk counterparts, exhibiting a band gap closure of around 1 eV upon the metal-off → metal-on transition, compared to only about 0.3 eV in the bulk. This behavior can be comprehensively justified by the presence of relative octahedral tilting in the quasi-2D metal-off structures, which is completely absent in the metal-on structures. Hence, the gap reduction results from a combination of both metal-on-centering and the suppression of relative octahedral tilting. In all remaining investigated systems, the reduction via the metal-off → metal-on transition is primarily due to the metal on-centering process and is therefore less drastic. For instance, in the conceived quasi-1D structures, the effect of the off → on transition on band gap narrowing is more modest, with *E*_g_ values changing from 3.64 to 3.47 eV for BA_3_PbI_5_ and from 3.25 to 3.12 eV for BA_3_SnI_5_.

From the band structures calculated through the DFT-1/2+SOC protocol (Figures S3 and S4) we have quantified the Rashba splitting effect along some paths in the Brillouin zone. The M ← R (X ← Γ) and R → Γ (Γ → Z) branches were employed for pseudocubic (tetragonal) as 3D structures, as well as X ← Γ and Γ → M for quasi-2D. For quasi-1D, we have considered only the periodic direction for the octahedral connections along X ← Γ. Figure 3 depicts the Rashba parameters (α = Δ*ε*/2Δ*k*) calculated through the *ε* wells vertices correlated with a particular *k*-points (Δ*k* = |*k* – *k*_0_|) for the valence (*ε*_V_) and conduction (*ε*_C_) bands; numerical details are available in Table S4 in Supporting Information.^13,45^

**Figure 3:**
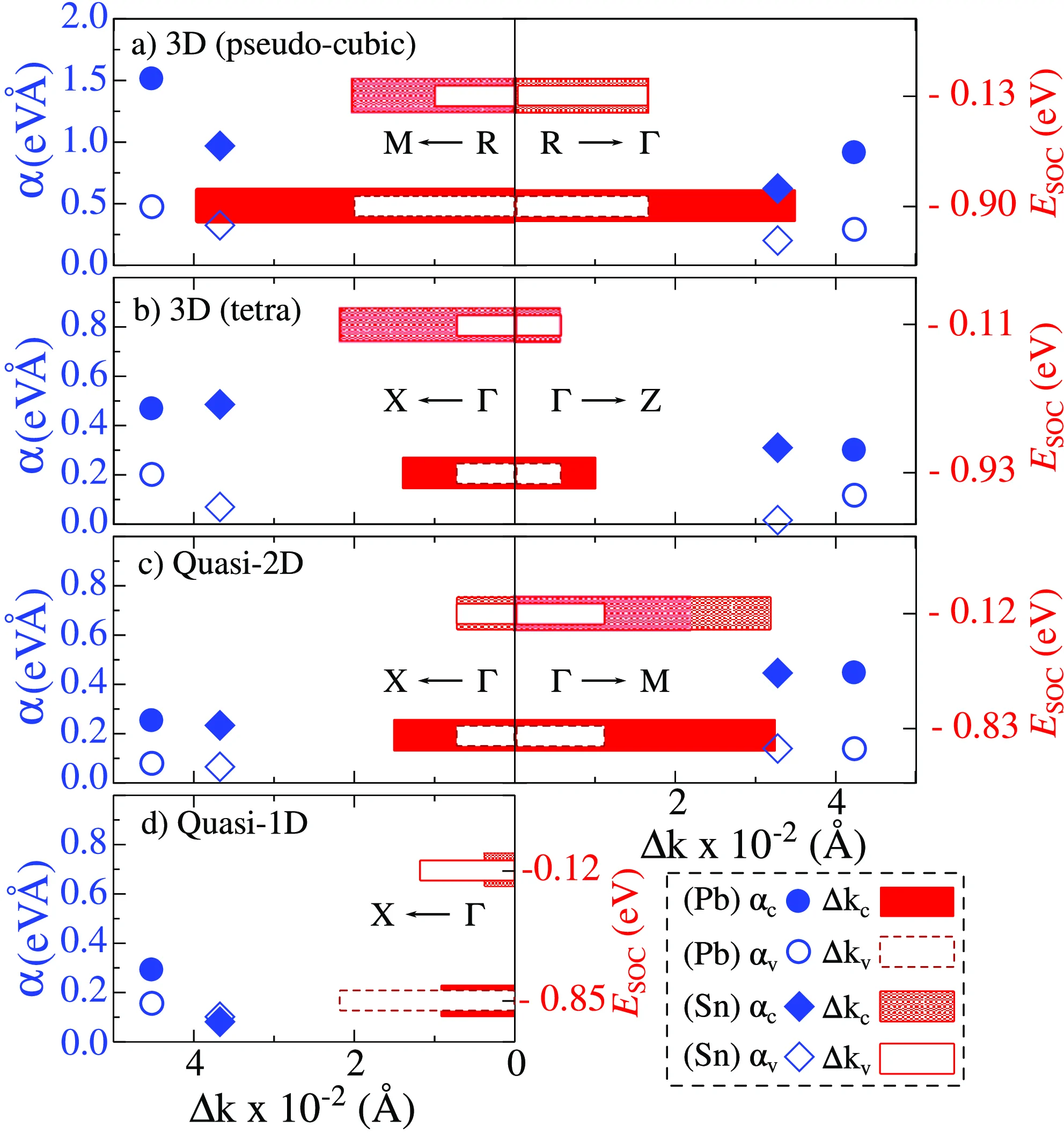
Rashba splitting parameters (blue) with respect to the Δ*k*, including SOC energies (red) calculated for the metal-off systems. The blue-filled circle and nonfilled circle show, respectively, the α_*c*_ and α_*v*_ values for the Pb-based systems, while the blue-filled and nonfilled diamonds show, respectively, the α_*c*_ and α_*v*_ values for the Sn-based systems. The red bars, each one with a different fill or line pattern, represent the Δ*k*_*c*_ and Δ*k*_*v*_ values, aligned with the SOC energies for Sn and Pb. Panels a), b), c), and d) refer, respectively, to 3D systems as pseudocubic, tetragonal, quasi-2D systems, and quasi-1D systems.

For all dimensionalities, one observes α_C_ ≥ α_V_, given the higher contributions of the metal-*p* orbital for the conduction band maximum associated with their SOC. For instance, for MAPbI_3_ pseudocubic (tetragonal) we found the highest α_C_ ∼ 1.52 eVÅ (∼0.46 eV Å) for the M ← R (X ← Γ) branch, where for MASnI_3_ α_C_ ∼ 0.99 eVÅ (∼0.49 eV Å) is the highest value, from which the tendencies based on α_p‑cub._ > α_tet._ are in agreement with previous bulk Rashba characterizations.^15^ Therefore, while one observes a suppression of pseudocubic → tetragonal changing due to the fitting of the MA^+^ cations into the cavities resulting from the out-of-phase rotation of the octahedra, which shortens the bond length deviations and approximates the metal to the octahedron center, for orthorhombic structures α is fully suppressed since the MA^+^ cations fit optimally for the in-phase octahedral rotations. As a result, the presenting bond-length asymmetries responsible for local inversion-symmetry breaking are eliminated, consequently suppressing the bulk Rashba splitting (α_v,c_ ∼ 0 eV Å).

Quasi-2D and model quasi-1D systems usually show the same pattern, where α_C_ is equal to or greater than α_V_. The only exception is BA_3_SnI_5_, where α_C_ is a bit smaller than α_V_. This happens because the quasi-1D structure has incomplete iodine coordination, which increases the iodine’s role at the top of the valence band. Compared to 3D structures, these lower-dimensional systems have smaller Rashba parameters. This arises from two distinct mechanisms. First, the BA^+^ spacer cations in the quasi-2D (and quasi-1D) systems exhibit characteristics that contrast with the 3D composition: with the NH_3_^+^ dipole moment oriented toward the layers, its dipole moment effect is mitigated, unlike in 3D systems, where dipole moments are aligned along a single direction. Second, the greater degrees of freedom within the octahedral network may favor the centering of the metal atom within the octahedron. Together, these two effects result in reduced distortion of the octahedral network and a smaller displacement of the metal atom from the center.

Still, the SOC contributions are significant: *E*_SOC_, the accumulated energy contribution arising from SOC, ranges from approximately −0.93 to −0.83 eV for Pb-based systems and from approximately −0.13 to −0.11 eV for Sn-based systems. The horizontal spin splitting, measured by Δ*k*_V_ and Δ*k*_C_, reflects local inversion-symmetry breaking more than just SOC strength. Overall, Δ*k*_C,V_(Pb) is greater than Δ*k*_C,V_(Sn), which matches the fact that Pb has stronger SOC. This also leads to another general trend, since the magnitude of the bulk Rashba effect is primarily determined by local distortions and SOC energies. At fixed symmetry, one observes that α_v,c(Pb)_ ≥ α_v,c(Sn)_, demonstrating how Rashba can be modulated according to composition.

Next, we evaluate the excitonic properties using the TB-BSE solution with Wannier Hamiltonians fitted to the DFT-1/2+SOC band structures (Figures S3 and S4 in the Supporting Information). Figure 4 presents typical exciton band structures. In the 3D systems, the exciton ground states are found at the high-symmetry point Γ in the pseudocubic, and also in tetragonal and orthorhombic structures, as shown in Figure S5. This result indicates that the lowest-energy excitons are bright.

**Figure 4:**
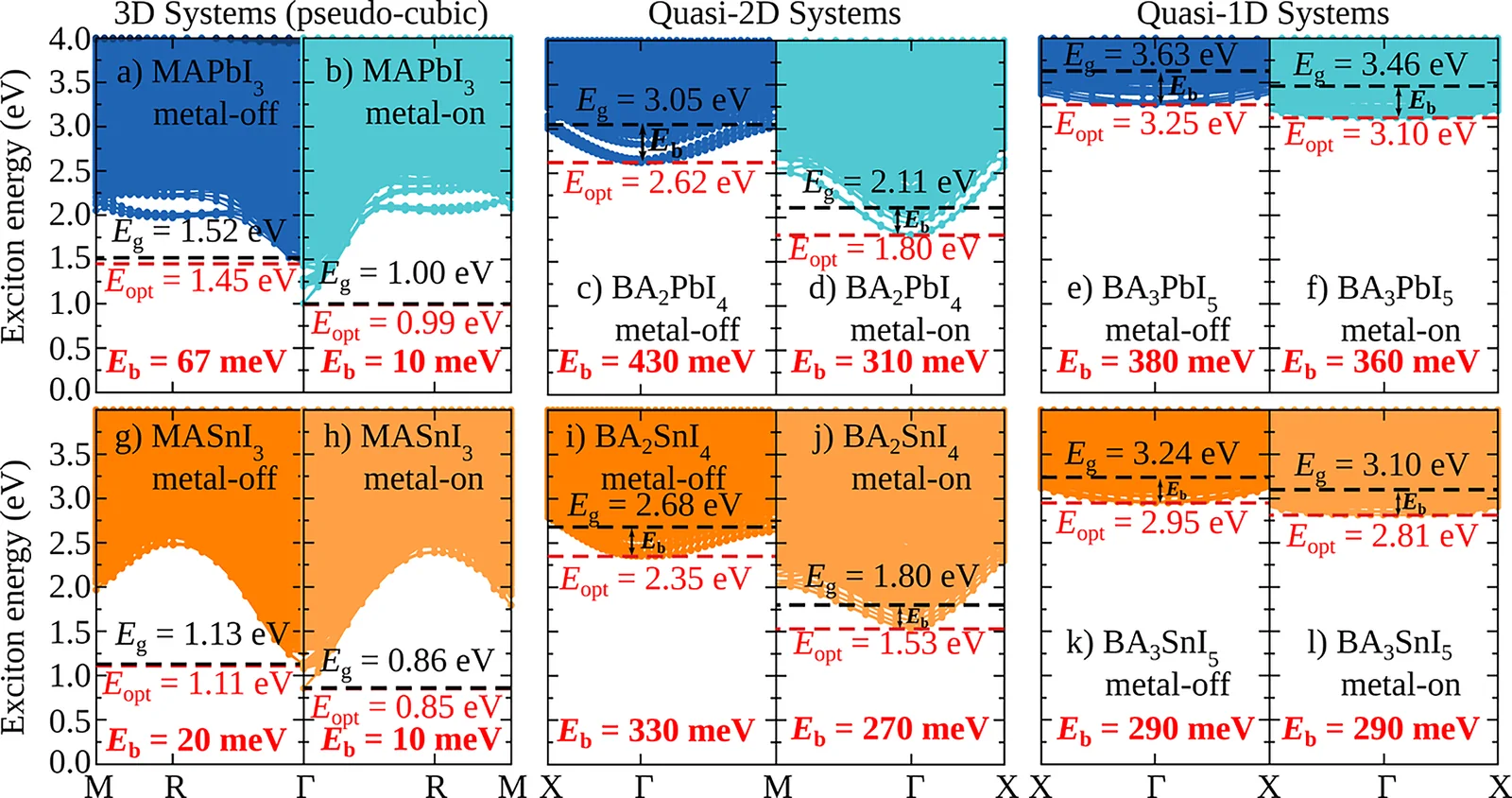
Calculated exciton band structures and exciton binding energies (*E*_b_) for representative systems of our investigation. The blue and cyan panels refer to the Pb-based systems, while the orange and light orange panels represent the Sn-based systems. The panels refer to a) 3D MAPbI_3_ metal-off, b) 3D MAPbI_3_ metal-on, c) quasi-2D BA_2_PbI_4_ metal-off, d) quasi-2D BA_2_PbI_4_ metal-on, e) quasi-1D BA_3_PbI_5_ metal-off, f) quasi-1D BA_3_PbI_5_ metal-on, g) 3D MASnI_3_ metal-off, h) 3D MASnI_3_ metal-on, (i) quasi-2D BA_2_SnI_4_ metal-off, j) quasi-2D BA_2_SnI_4_ metal-on, k) quasi-1D BA_3_SnI_5_ metal-off, l) quasi-1D BA_3_SnI_5_ metal-on.

Dimensional confinement leads to the largest increase in exciton binding as a dominant effect for the correlation between structure and *E*_b_ magnitude. For instance, in quasi-2D systems, *E*_b_ = 430 meV for BA_2_PbI_4_ and 330 meV for BA_2_SnI_4_, both much higher than the 3D as pseudocubic, tetragonal, and orthorhombic *E*_b_ values. On the other hand, the metal-off → metal-on transformation yields an increment on the *E*_b_ decreasing as a secondary effect, since these values drop to 310 and 270 meV Pb- and Sn-based quasi-2D systems. This effect is also observed for the 3D phases throughout the crystallographic symmetry increasing from orthorhombic to tetragonal to pseudocubic, i.e., *E*_b_ decreases. For MAPbI_3_, *E*_b_ drops from 240 meV in the orthorhombic phase to 220 meV in the tetragonal phase, and then to 67 meV in the pseudocubic phase, whereas for MASnI_3_, the values follow a similar trend: 190, 160, and 20 meV. Advancing for the metal-off → metal-on transition, since for orthorhombic systems the metal-off and -on structures are equivalent and *E*_b_ does not change, for tetragonal MAPbI_3_ (MASnI_3_), *E*_b_ reduces to 180 meV (150 meV), whereas for pseudocubic reduces to 10 meV (10 meV).

The Rashba suppression modifies the band edges, which are associated with the spin nature of the charge carriers. With significant Rashba splitting, the valence band maximum (VBM) and conduction band minimum (CBM) display opposite spin textures, resulting in a (slower) spin-forbidden radiative recombination transition. Once the Rashba effect is weakened, VBM and CBM now exhibit aligned spin textures, leading to a (faster) spin-allowed recombination dynamics. Our results complement this mechanism, now associating the switch from spin-forbidden to spin-allowed transitions with a decrease in exciton binding energies. Moreover, it is important to mention that the observed reduction in exciton binding energy accompanying Rashba suppression should be interpreted as part of a broader modification of the electronic structure rather than as evidence of a direct causal relationship, since effective masses, band-edge dispersion, orbital localization, and dielectric screening are all expected to evolve simultaneously. Consequently, the present calculations do not allow these individual contributions to be quantitatively disentangled. For instance, the theoretical quasi-1D systems also have stronger binding than their 3D counterparts, with *E*_b_ = 380 meV for BA_3_PbI_5_ and 290 meV for BA_3_SnI_5_. However, *E*_b_ does not continue to increase from quasi-2D to quasi-1D, given the last ones are slightly lower by showing that confinement is not the only factor affecting binding energy.

The lower *E*_b_ values in Sn-based systems compared to Pb-based ones match their different SOC strengths and band-edge characters, but this difference should not be seen as a direct Coulomb effect of SOC alone. Table S5 in the Supporting Information presents the calculated electron and hole effective masses. The reduced effective masses follow the same qualitative trends observed for the exciton binding energies, including the decrease from metal-off to metal-on configurations and the larger values found in low-dimensional systems. However, the effective masses alone do not fully account for the observed variations in the *E*_b_. In particular, the quasi-2D and model quasi-1D systems exhibit similar reduced masses while displaying different exciton binding energies, indicating that dielectric screening, orbital localization, and band edge dispersion also contribute significantly. Therefore, the effective mass analysis supports the observed qualitative trends but does not uniquely determine the exciton binding energies.

We further analyzed the linear optical response through the absorption coefficients for x̂ , ŷ , and ẑ polarizations (Figure 5 and Figure S6 in the Supporting Information). The spectra were evaluated from 0.0 to 2.0 eV for the 3D systems and from 0.0 to 4.0 eV for the quasi-2D and quasi-1D systems. Comparing IPA and BSE spectra shows that excitonic effects red shift the optical onset in all dimensionalities. For pseudocubic MAPbI_3_ and MASnI_3_, the metal-off → metal-on transformation makes the optical response more isotropic and strongly reduces the separation between *E*_g_ and *E*_opt_. This behavior follows from the restoration of local metal-centered symmetry and the associated modification of band-edge hybridization.

**Figure 5:**
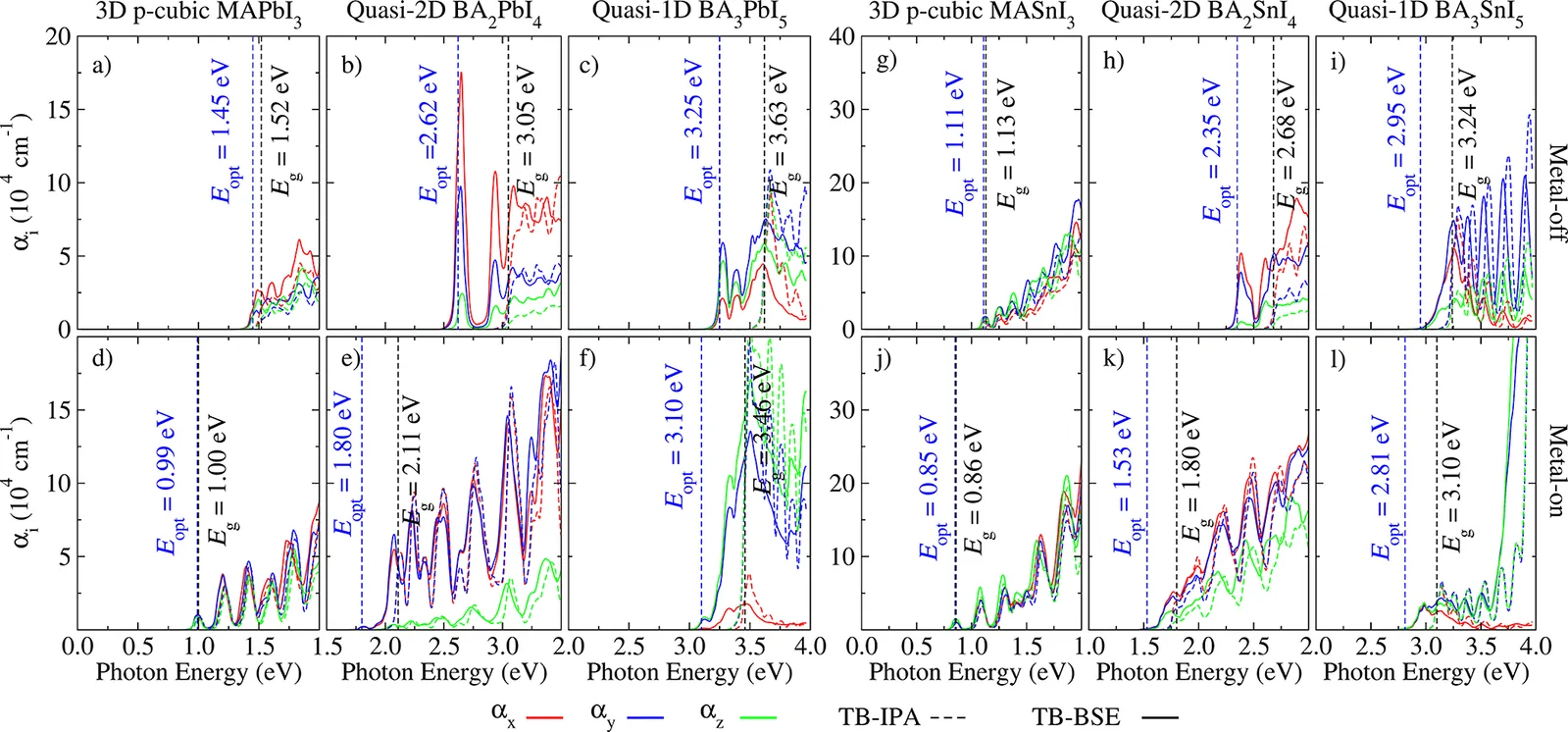
Absorption coefficient (α) calculated through independent particle approximation ((W)­TB+IPA as dashed lines) and through (W)­TB+BSE formalism (solid lines) for the excitonic effects. The red, blue, and green colors represent α along the *x*-, *y*-, and z-axes. The values of electronic band gap (*E*_g_) and optical band gap (*E*_opt_) are depicted in each graphic. The first-row panels depict metal-off structures, while the second-row panels depict metal-on structures. The panels refer to a) 3D MAPbI_3_ metal-off, b) quasi-2D BA_2_PbI_4_ metal-off, c) quasi-1D BA_3_PbI_5_ metal-off, d) 3D MAPbI_3_ metal-on, e) quasi-2D BA_2_PbI_4_ metal-on, f) quasi-1D BA_3_PbI_5_ metal-on, g) 3D MASnI_3_ metal-off, h) quasi-2D BA_2_SnI_4_ metal-off, (i) quasi-1D BA_3_SnI_5_ metal-off, j) 3D MASnI_3_ metal-on, k) quasi-2D BA_2_SnI_4_ metal-on, l) quasi-1D BA_3_SnI_5_ metal-on.

In quasi-2D BA_2_PbI_4_ and BA_2_SnI_4_, the in-plane components α_*x*_ and α_*y*_ are the main contributors to absorption near the optical onset. The out-of-plane response α_*z*_ depends strongly on the direction of the organic spacer. Figure S7 in the Supporting Information shows the project density of states (PDOS) for our low-dimensionality systems. For the hypothetical quasi-1D systems BA_3_PbI_5_ and BA_3_SnI_5_, the optical response is highly anisotropic and stays that way after the metal-off to metal-on transformation, which matches the wire-like structure of the inorganic framework. One way to explain this α_*i*_ anisotropy in quasi-1D systems is to analyze the contributions of iodine atoms along the wire axis versus those extending beyond the wire periodicity, using the PDOS for localized iodine atoms, as shown in Figure S8 in the Supporting Information. Interestingly, the iodine atoms located outside the periodicity of the wire are found to contribute much more significantly to the electronic states at the valence band-edge, which explains why α_*z*_ and α_*y*_dominates so strongly over the in-plane components in these systems. The calculated photoluminescence spectra at 300 K (Figures S9 and S10 in the Supporting Information) have emission peaks that match *E*_opt_, which confirms that the lowest emissive excitons are bright.

Our results show that dimensionality, polymorphism, and local metal off-centering influence excitonic effects in Pb- and Sn-based iodide metal halide perovskites. We used DFT-1/2+SOC band structures, mapped onto Wannier tight-binding Hamiltonians, and solved the resulting Hamiltonians using the Bethe–Salpeter equation. In 3D polymorphs, we found a clear trend: the exciton binding energy (*E*_b_) decreases from orthorhombic to tetragonal to pseudocubic structures. When composition and dimensionality are fixed, changing from metal-off to metal-on restores local metal-centered symmetry, reduces Rashba splitting, and generally lowers the exciton binding energy. Our findings demonstrate that dimensional confinement remains as the dominant factor governing exciton binding energies. Nevertheless, the metal-off → metal-on transition at fixed composition and dimensionality plays a secondary role by modifying the band-edge electronic structure. However, Rashba-active distortions should be interpreted as part of a broader electronic structure modification involving band-edge dispersion, effective masses, orbital character, and dielectric screening, all of which contribute to the observed excitonic behavior. Quasi-2D BA_2_PbI_4_/BA_2_SnI_4_ and theoretical quasi-1D BA_3_PbI_5_/BA_3_SnI_5_ have much larger *E*_b_ than their 3D counterparts. However, the quasi-1D values are not higher than the quasi-2D values, which means that confinement, orbital character, and band dispersion all play a role. Our calculated photoluminescence spectra also show that the lowest-emission excitons appear at *E*_opt_ across all the systems we studied.

## Supplementary Material



## Notes

Extra results for the geometric and electronic properties (PDF)
