# Supplementary material for: Correlating Rashba Suppression with Exciton Binding in Low-Dimensional Metal Halide Perovskites
Source: J Phys Chem Lett. 2026 Jul 29;17(39):11039–46. doi: 10.1021/acs.jpclett.6c01964 (PMC13637077; doi:10.1021/acs.jpclett.6c01964)
Supplement: Supplementary file 1 [file jz6c01964_si_001.pdf]

Supporting Information: Correlating Rashba Suppression with Exciton Binding in Low-Dimensional Metal Halide Perovskites

Marco Antonio de Andrade Queiroz,[*,†] Danilo Neves Silveira,[†] Fernando P. Sabino,[‡] Carlos M. O. Bastos,[¶,§] Celso R. C. Rêgo,[‖] Maurício J. Piotrowski,[⊥] Alexandre C. Dias,[¶,§] and Diego Guedes-Sobrinho[*,†]

†*Quantum Chemistry and Thermodynamic Materials Group, Department of Chemistry, Federal University of Paraná, CEP 81531-980, Curitiba, Brazil*

‡*Materials Engineering Department, Engineering School of São Carlos, University of São Paulo, CEP 13566-590, São Carlos, Brazil*

¶*Institute of Physics and International Center for Physics, University of Brasília, Brasília, DF 70919-970, Brazil*

§*Computational Materials Laboratory, LCCMat, Institute of Physics, University of Brasília, 70910-900, Brasília, Brazil.*

‖*Institute of Nanotechnology Hermann-von-Helmholtz-Platz, Karlsruhe Institute of Technology, 76021 Karlsruhe, Germany*

⊥*Department of Physics, Federal University of Pelotas, Pelotas, RS 96010-900, Brazil*

E-mail: marco.queiroz@ufpr.br; guedes.sobrinho.d@gmail.com

I DFT-1/2 Quasiparticle Correction Method

The electronic characterization, mainly the band gap determination, was made possible through the DFT-1/2 quasiparticle correction method, know for its low computational cost and excellent agreement with experimental data. This method stems from the Slater transition technique, accounting for the known gap underestimation problem through the half electron/half hole occupation, whose atomic eigenvalue is equal to the atomic ionization potential. Its application was extended to periodic systems by Ferreria et al., where the half occupation is now associated with a modified Kohn-Sham potential as $V_{\mathrm{mod,KS}} = V_{\mathrm{KS}}(\vec{r}) - V_S(\vec{r})$, with $V_{\mathrm{KS}}(\vec{r})$ being the standard Kohn-Sham potential and $V_S(\vec{r})$ the self-energy potential. $V_S(\vec{r})$, depicted in Equation 1, is calculated through the energy difference between the KS potentials for neutral (all-electron, ae) and half-ionized (ae-1/2) atoms. It also needs to be trimmed by a function $\Theta(\vec{r}, CUT)$, or the interpenetration of the self-energy Coulomb tails into neighboring atom sites may occur.

$$V_S(\vec{r}) = \Theta(\vec{r}, CUT)[V_{ae}(\vec{r}) - V_{ae-1/2}(\vec{r})]. \tag{1}$$

The *CUT* parameter is determined variationally through the gap energy's maximization. No empirical parameters are taken into consideration.

We performed the gap energy (E_{g}) calculations for all pure perovskites: 3D systems (pseudo-cubic, tetragonal and orthorhombic), quasi-2D systems and quasi-1D systems. We also performed these calculations for the 3D (pseudo-cubic and tetragonal), quasi-2D and quasi-1D systems considering centrossymetric platonic-like octahedrons, systems named as metal-on along this article. This was not necessary for the 3D orthorhombic structures, due to their natural centrossymetric octahedrons. The character of the top of valence band (all systems) and conduction band (quasi-2D and quasi-1D systems) was analyzed to define the potential to be corrected. The *CUT* parameter determination for each system is depicted in Figure S1. The values for each system are: $MAPbI_3$ (all phases) - 3.84a_0, $MASnI_3$ (all phases) - 3.62a_0, BA_2PbI_4 - 3.88a_0 (valence) and 4.76a_0 (conduction), BA_2SnI_4 - 3.74a_0 (valence) and 4.80a_0 (conduction), BA_3PbI_5 - 4.22a_0 (valence) and 5.00a_0

(conduction), BA_3SnI_5 - 4.20a_0 (valence) and 5.00a_0 (conduction). The same values were applied to the metal-on systems, due to the good transferability of the DFT-1/2 method.

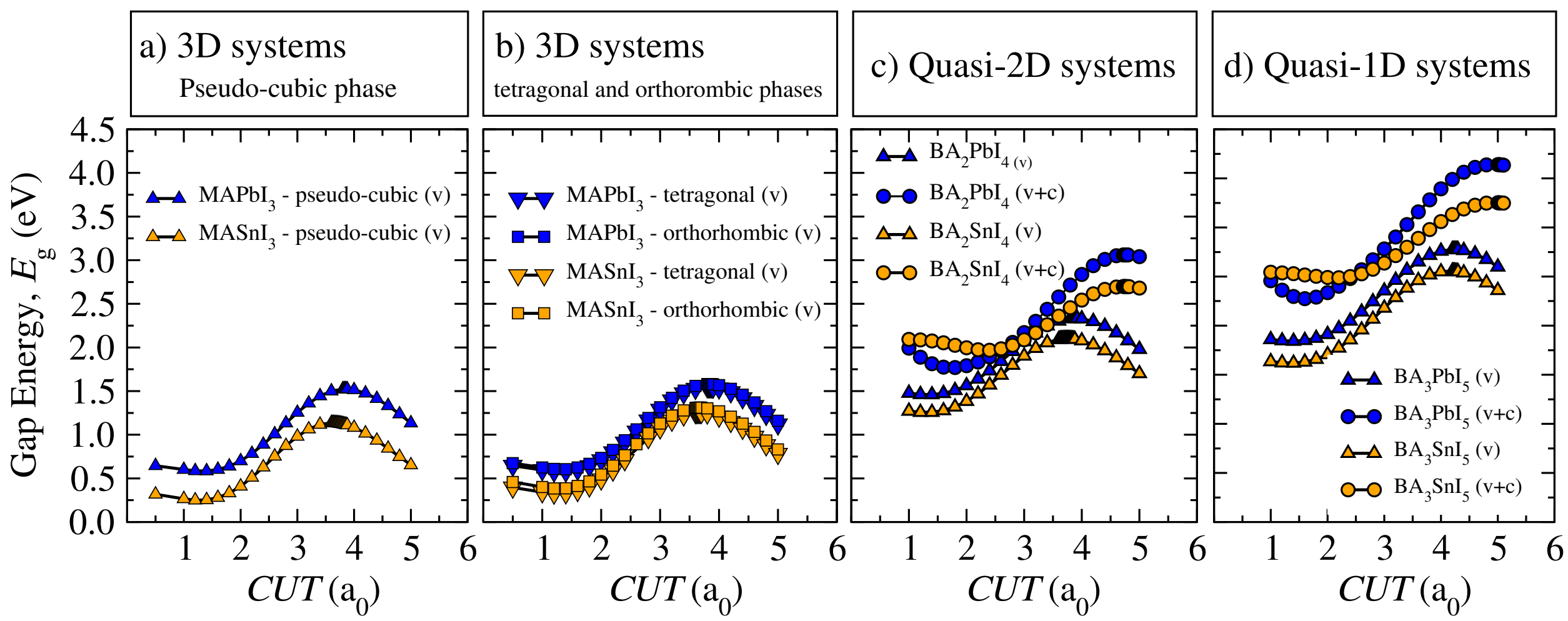

Figure S1. Optimization of the CUT parameter for a) 3D systems in pseudo-cubic phase, b) 3D systems in tetragonal and orthorhombic phases, c) Quasi-2D systems and d) Quasi-1D systems, in the Bohr radius a_0, within our protocol DFT-1/2+SOC through half-ionization around the valence band for the 5p state from the I and around the conduction band for the 6p state from the Pb or the 5p state from the Sn.

II WanTiBEXOS Code Details

Table S1. Wantibexos parameters employed throughout the excitonic properties (exciton band structures, optical absorption coefficient and photoluminescence) calculations. The parameters are the BSE Grid, number of conduction bands (nc), number of valence bands (nv) and Coulombic Potential. The first ten lines refer to the 3D systems, the next four lines refer to the quasi-2D systems and the last four lines refer to the quasi-1D systems.

System	BSE Grid.			nc	nv	Coul. Pot
**3D Systems**						
3D - $MAPbI_3$ (p-cub. met-off)	14	14	14	6	6	V3D
3D - $MAPbI_3$ (p-cub. met-on)	14	15	14	6	6	V3D
3D - $MAPbI_3$ (tet. met-off)	16	16	11	6	6	V3D
3D - $MAPbI_3$ (tet. met-on)	16	16	11	6	6	V3D
3D - $MAPbI_3$ (ort.)	17	11	15	6	6	V3D
3D - $MASnI_3$ (p-cub. met-off)	14	14	14	6	6	V3D
3D - $MASnI_3$ (p-cub. met-on)	14	15	14	6	6	V3D
3D - $MASnI_3$ (tet. met-off)	16	16	11	6	6	V3D
3D - $MASnI_3$ (tet. met-on)	16	16	11	6	6	V3D
3D - $MASnI_3$ (ort.)	17	11	15	6	6	V3D
**Quasi-2D Systems**						
2D - BA_2PbI_4 (met-off)	19	19	11	2	2	V3D
2D - BA_2PbI_4 (met-on)	19	19	11	4	6	V3D
2D - BA_2SnI_4 (met-off)	19	19	11	4	6	V3D
2D - BA_2SnI_4 (met-on)	19	19	11	4	6	V3D
**Quasi-1D Systems**						
1D - BA_3PbI_5 (met-off)	13	11	11	4	2	V3D
1D - BA_3PbI_5 (met-on)	13	11	11	16	2	V3D
1D - BA_3SnI_5 (met-off)	13	11	11	4	2	V3D
1D - BA_3SnI_5 (met-on)	13	11	11	16	4	V3D

III Structural Parameters

Table S2. Lattice parameters, experimental lattice parameters with percentage deviations, bond distances, bond angles and unit cell volumes for all the calculated systems. The first ten lines exhibit the 3D systems structural values, the next four lines refer to the quasi-2D systems and the last four lines refer to the quasi-1D systems.

	Lat. Param. [Å]			Exp. Lat. Param. [Å]			M-I Bonds (Å)		M-I-M Bonds (°)		Volume (Å^3)
System	a	b	c	a	b	c	Equatorial	Apical	Equatorial	Apical	
**3D Systems**											
3D - MAPbI$_3$ p-cub. met-off	6.40	6.33	6.44	6.28 (1.9 %)	6.28 (0.8 %)	6.28[1] (2.5 %)	3.07-3.34	3.20-3.26	167-171	170-170	260.48
3D - MAPbI$_3$ p-cub. met-on	6.26	6.19	6.29	x	x	x	3.09-3.13	3.14-3.14	180-180	180-180	243.75
3D - MAPbI$_3$ tet. met-off	8.75	8.75	12.78	8.79 (−0.5 %)	8.79 (−0.5 %)	12.55[1] (1.8 %)	3.16-3.25	3.17-3.23	151-151	174-176	977.85
3D - MAPbI$_3$ tet. met-on	8.74	8.75	12.78	x	x	x	3.15-3.15	3.19-3.19	157-157	180-180	977.85
3D - MAPbI$_3$ ort.	8.42	12.57	9.11	x	x	x	3.16-3.16	3.21-3.21	151-151	165-165	964.10
3D - MASnI$_3$ p-cub. met-off	6.30	6.23	6.33	6.23 (1.1 %)	6.23 (0.0 %)	6.23[2] (1.6 %)	2.97-3.35	3.12-3.12	170-173	170-170	248.15
3D - MASnI$_3$ p-cub. met-on	6.30	6.23	6.33	x	x	x	3.09-3.13	3.14-3.14	180-180	180-180	243.75
3D - MASnI$_3$ tet. met-off	8.75	8.75	12.78	8.76 (−0.1 %)	8.76 (−0.1 %)	12.43[2] (2.8 %)	3.02-3.30	3.10-3.29	157-157	172-176	977.85
3D - MASnI$_3$ tet. met-on	8.75	8.75	12.78	x	x	x	3.15-3.15	3.19-3.19	157-157	180-180	977.85
3D - MASnI$_3$ ort.	8.42	12.56	9.11	x	x	x	3.16-3.16	3.18-3.18	156-168	156-168	964.10
**Quasi-2D Systems**											
2D - BA$_2$PbI$_4$ met-off	8.34	8.90	15.38	8.43 (-1.0%)	8.99 (-1.0%)	26.23[3] (-41.4%)	3.15-3.26	3.21-3.26	139-150	x-x	1059.33
2D - BA$_2$PbI$_4$ met-on	8.34	8.90	15.38	x	x	x	3.02-3.07	3.06-3.06	180-180	x-x	1059.33
2D - BA$_2$SnI$_4$ met-off	8.34	8.90	15.38	8.44 (-1.2%)	8.94 (-0.4%)	26.17[4] (-41.2%)	3.10-3.27	3.14-3.23	141-150	x-x	1059.33
2D - BA$_2$SnI$_4$ met-on	8.34	8.90	15.38	x	x	x	3.02-3.07	3.06-3.06	180-180	x-x	1059.33
**Quasi-1D Systems**											
1D - BA$_3$PbI$_5$ met-off	14.00	16.00	16.00	x	x	x	3.08-3.61	3.10-3.51	173-173	x-x	3584.00
1D - BA$_3$PbI$_5$ met-on	14.00	16.00	16.00	x	x	x	3.50-3.50	3.50-3.50	180-180	x-x	3584.00
1D - BA$_3$SnI$_5$ met-off	14.00	16.00	16.00	x	x	x	3.08-3.61	3.10-3.51	173-173	x-x	3584.00
1D - BA$_3$SnI$_5$ met-on	14.00	16.00	16.00	x	x	x	3.50-3.50	3.50-3.50	180-180	x-x	3584.00

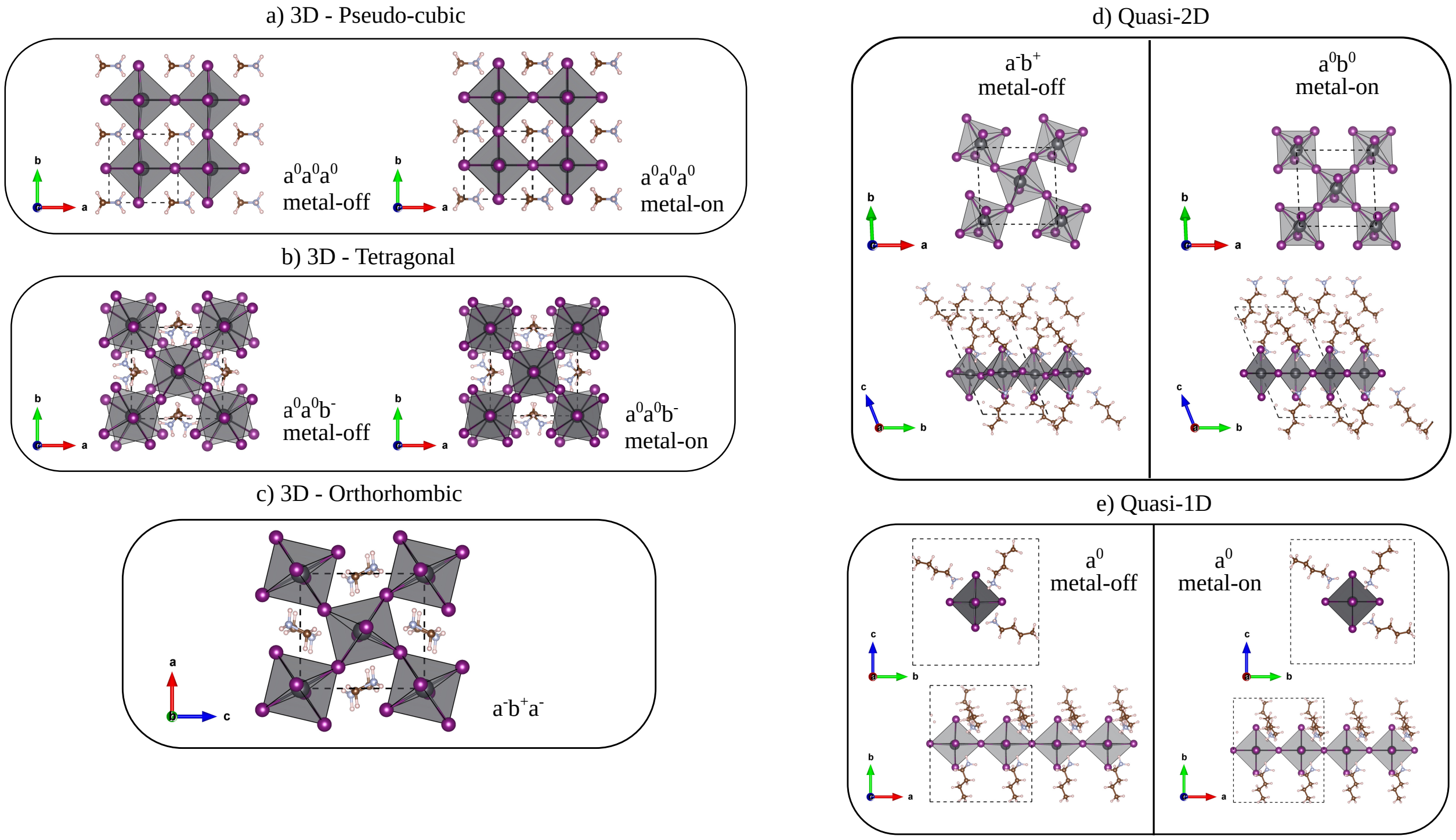

Figure S2. Representations of the systems' structures, along with their respective Glazer notations and metal-on/metal-off classifications. The dashed black lines represent the system's unit cells. The panels refer to a) $MAMI_3$ in pseudo-cubic phase; b) $MAMI_3$ in tetragonal phase; c) $MAMI_3$ in orthorhombic phase; d) quasi-2D BA_2MI_4 and e) quasi-1D BA_3MI_5 (M = Pb^{2+}, Sn^{2+}).

IV Electronic Structure, Wannierization and Bulk Rashba

Table S3. Gap energies obtained through our DFT-1/2+SOC protocol in comparison with available experimental data and percentage deviations. The first ten lines refer to the 3D systems, the next four lines refer to the quasi-2D systems and the last four lines refer to the quasi-1D systems.

System	Gap energy (eV)	Exp. (eV)	Dev. (%)
**3D Systems**			
3D - $MAPbI_3$ (p-cub. met-off)	1.52	1.48	2.7
3D - $MAPbI_3$ (p-cub. met-on)	1.01	–	–
3D - $MAPbI_3$ (tet. met-off)	1.54	1.52	1.3
3D - $MAPbI_3$ (tet. met-on)	1.34	–	–
3D - $MAPbI_3$ (ort.)	1.57	1.67	-5.9
3D - $MASnI_3$ (p-cub. met-off)	1.13	1.33	-15.0
3D - $MASnI_3$ (p-cub. met-on)	0.86	–	–
3D - $MASnI_3$ (tet. met-off)	1.25	1.21	3.3
3D - $MASnI_3$ (tet. met-on)	1.19	–	–
3D - $MASnI_3$ (ort.)	1.30	–	–
**Quasi-2D Systems**			
2D - BA_2PbI_4 (met-off)	3.06	2.56	19.5
2D - BA_2PbI_4 (met-on)	2.09	–	–
2D - BA_2SnI_4 (met-off)	2.69	2.18	23.3
2D - BA_2SnI_4 (met-on)	1.78	–	–
**Quasi-1D Systems**			
1D - BA_3PbI_5 (met-off)	3.64	–	–
1D - BA_3PbI_5 (met-on)	3.47	–	–
1D - BA_3SnI_5 (met-off)	3.25	–	–
1D - BA_3SnI_5 (met-on)	3.12	–	–

Experimental values from Refs.[2,5–10].

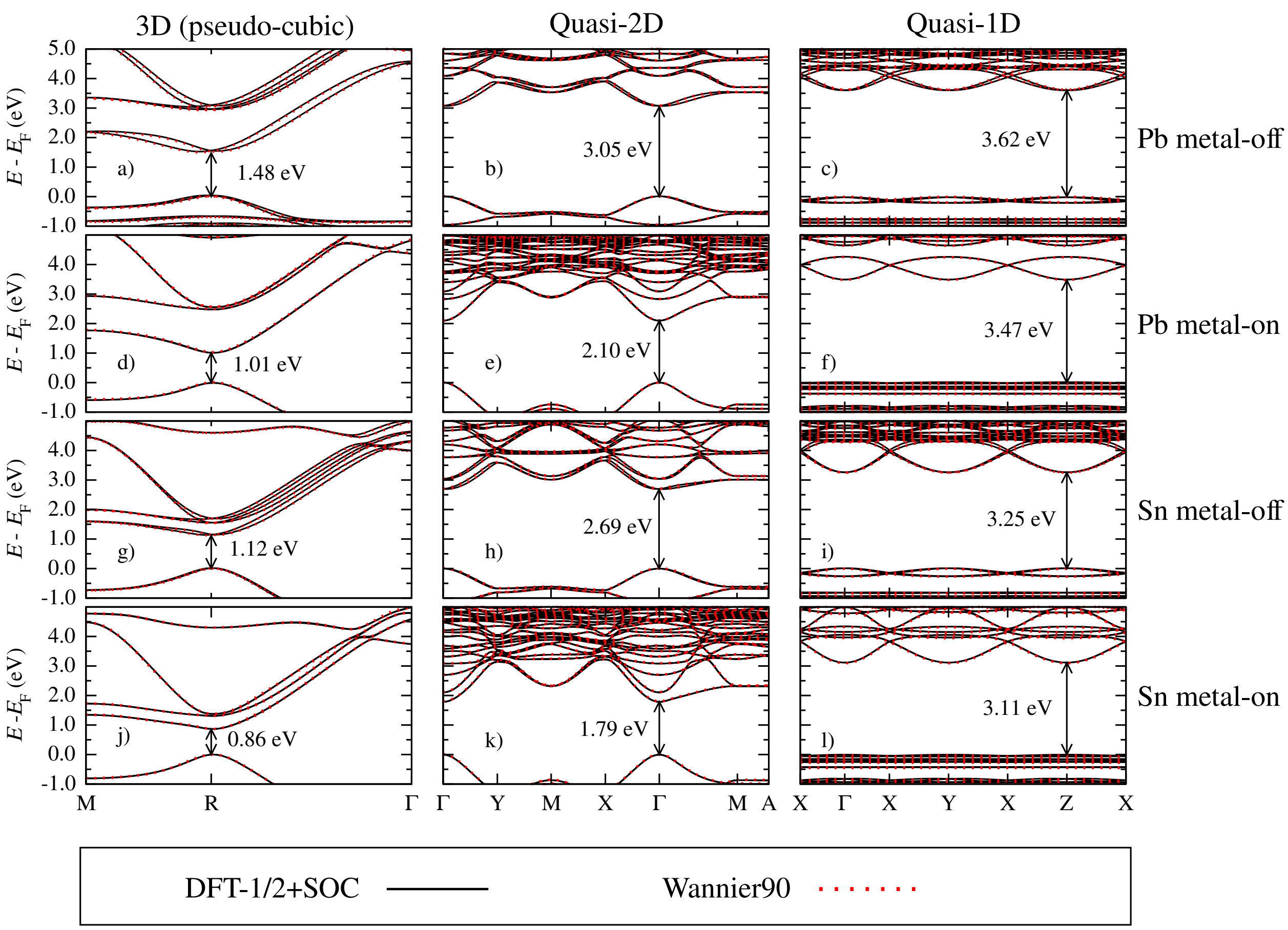

Figure S3. Band structures determined with DFT-1/2 (black lines) and with Wannier90 (red dashed lines), showing a perfect match for all the systems. First column refers to 3D pseudo-cubic systems, second column refers to Quasi-2D systems and the third column refers to Quasi-1D systems. The first and third rows refer to Pb and Sn metal-off systems, respectively, and the second and fourth rows refer to metal-on Pb and Sn systems, respectively. The panels are: a) 3D $MAPbI_3$ pseudo-cubic metal-off, b) quasi-2D BA_2PbI_4 metal-off, c) quasi-1D BA_3PbI_5 metal-off, d) $MAPbI_3$ pseudo-cubic metal-on, e) quasi-2D BA_2PbI_4 metal-on; f) quasi-1D BA_3PbI_5 metal-on; g) 3D $MASnI_3$ pseudo-cubic metal-off; h) quasi-2D BA_2SnI_4 metal-off; i) quasi-1D BA_3SnI_5 metal-off; j) $MASnI_3$ pseudo-cubic metal-on; k) quasi-2D BA_2SnI_4 metal-on; l) quasi-1D BA_3SnI_5 metal-on.

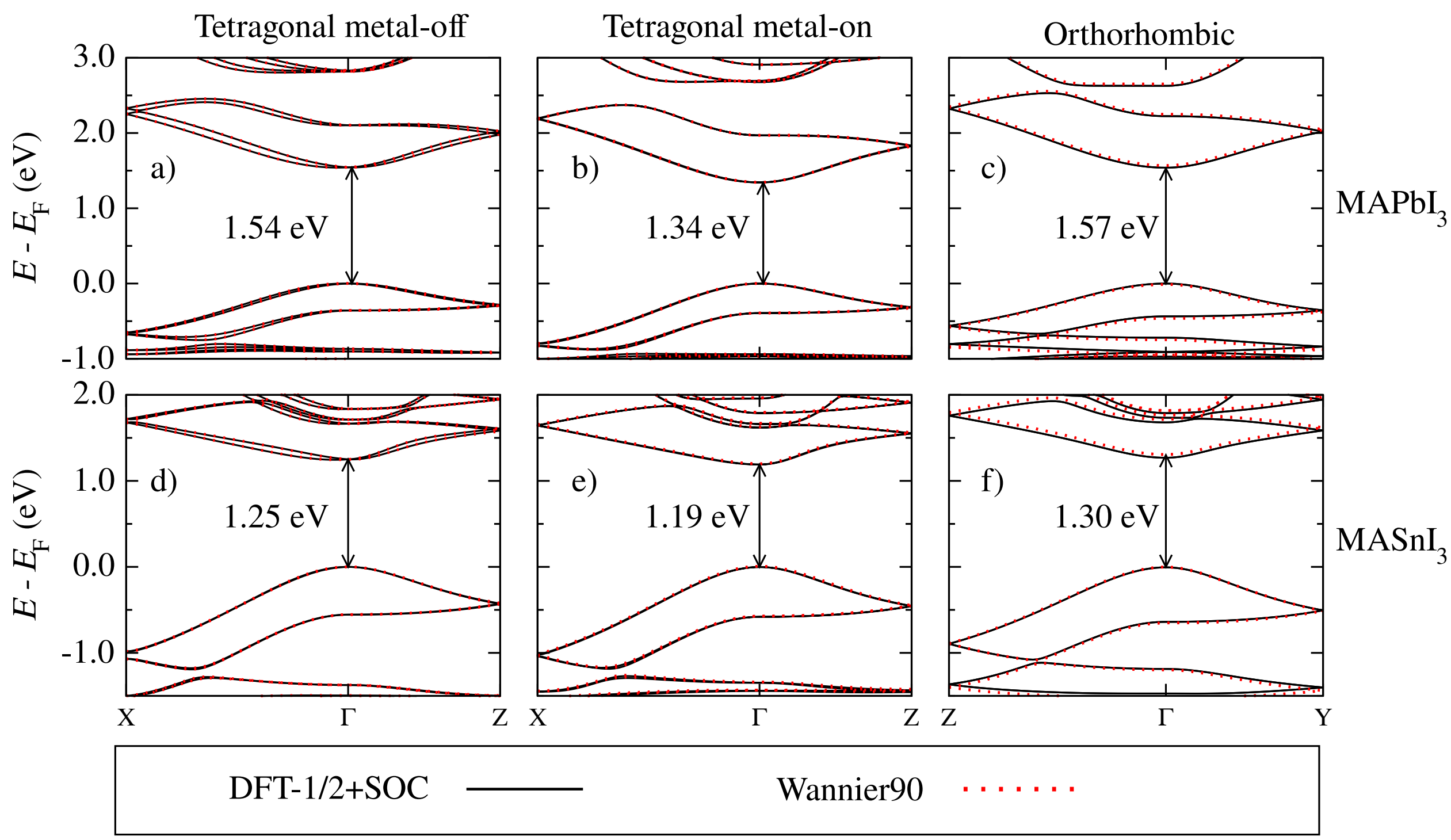

Figure S4. Band structures determined with DFT-1/2 (black lines) and with Wannier90 (red dashed lines), showing a perfect match for all the systems. First column refers to 3D tetragonal metal-off systems, second column refers to tetragonal metal-on systems and the third column refers to orthorhombic systems. The first row refers to Pb systems, and the second row refers to Sn systems, respectively. The panels are: a) $MAPbI_3$ tetragonal metal-off; b) $MAPbI_3$ tetragonal metal-on; c) $MAPbI_3$ orthorhombic; d) $MASnI_3$ tetragonal metal-off; e) $MASnI_3$ tetragonal metal-on; f) $MASnI_3$ orthorhombic.

Table S4. **α parameters and Δk values through different branches, along with SOC energies relative to Pb and Sn. First two lines refer to the values obtained for $MAPbI_3$ and $MASnI_3$ in pseudo-cubic phase. Next two lines refer to the same systems in tetragonal phase. Next two lines refer to quasi-2D systems BA_2PbI_4 and BA_2SnI_4, and the last 2 lines refer to quasi-1D systems BA_3PbI_5 and BA_3SnI_5.**

**3D Systems (pseudo-cubic)**										
	α (M ← R) (eVÅ)		Δk (M ← R) (10^{-2} Å)		α (R → Γ) (eVÅ)		Δk (R → Γ) (10^{-2} Å)		E_{SOC} (meV)	
System	α_v	α_c	Δk_v	Δk_c	α_v	α_c	Δk_v	Δk_c	Pb	Sn
$MAPbI_3$	0.46	1.52	2.0	4.0	0.28	0.92	1.7	3.4	-0.90	x
$MASnI_3$	0.30	0.99	1.0	2.0	0.17	0.64	1.7	1.7	x	-0.13
**3D Systems (tetragonal)**										
	α (X ← Γ) (eVÅ)		Δk (X ← Γ) (10^{-2} Å)		α (Γ → Z) (eVÅ)		Δk (Γ → Z) (10^{-2} Å)		E_{SOC} (meV)	
System	α_v	α_c	Δk_v	Δk_c	α_v	α_c	Δk_v	Δk_c	Pb	Sn
$MAPbI_3$	0.20	0.46	0.7	1.4	0.13	0.31	0.5	1.0	-0.93	x
$MASnI_3$	0.08	0.49	0.7	2.2	0.01	0.33	0.5	0.5	x	-0.11
**Quasi-2D Systems**										
	α (X ← Γ) (eVÅ)		Δk (X ← Γ) (10^{-2} Å)		α (Γ → M) (eVÅ)		Δk (Γ → M) (10^{-2} Å)		E_{SOC} (meV)	
System	α_v	α_c	Δk_v	Δk_c	α_v	α_c	Δk_v	Δk_c	Pb	Sn
BA_2PbI_4	0.07	0.25	0.7	1.5	0.13	0.45	1.1	3.3	-0.83	x
BA_2SnI_4	0.06	0.24	0.7	0.7	0.13	0.45	1.1	3.3	x	-0.12
**Quasi-1D Systems**										
	α (X ← Γ) (eVÅ)		Δk (X ← Γ) (10^{-2} Å)		E_{SOC} (meV)					
System	α_v	α_c	Δk_v	Δk_c	Pb	Sn				
BA_3PbI_5	0.18	0.30	2.2	0.9	-0.85	x				
BA_3SnI_5	0.12	0.10	1.3	0.4	x	-0.12				

V Optoelectronic Properties

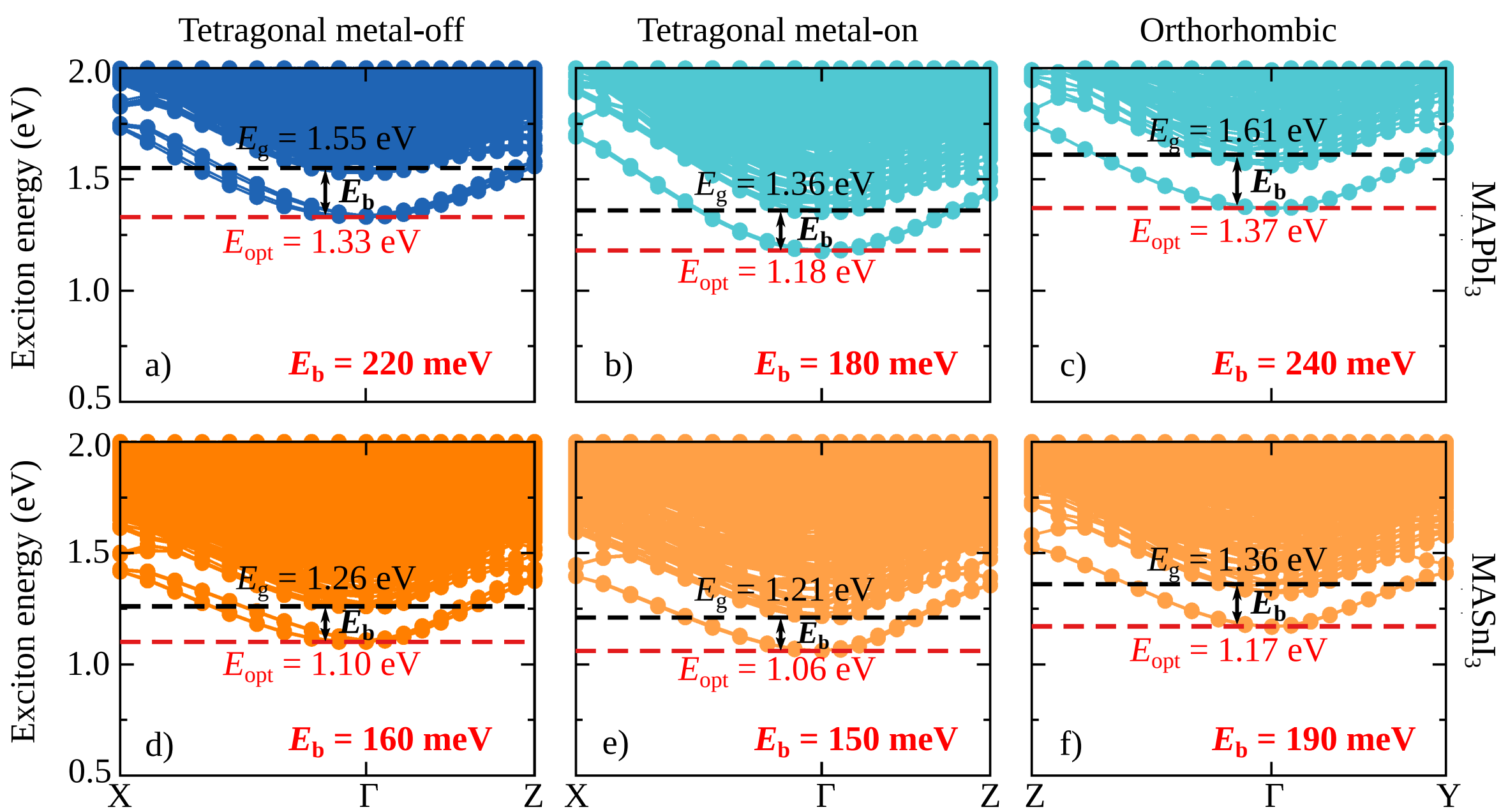

Figure S5. Calculated exciton band structures and exciton binding energies (E_b) for our 3D systems considering tetragonal and orthorhombic phases. The blue and cyan panels refer to the $MAPbI_3$ structures, whilst the orange and light orange panels refer to the $MASnI_3$ structures. The panels represent: a) $MAPbI_3$ tetragonal metal-off, b) $MAPbI_3$ tetragonal metal-on, c) $MAPbI_3$ orthorhombic, d) $MASnI_3$ tetragonal metal-off, e) $MASnI_3$ tetragonal metal-on, f) $MASnI_3$ orthorhombic.

Table S5. Calculated effective masses of electron and hole (in units of free electron mass) through our DFT-1/2+SOC protocol.

System	m_h^*			m_e^*		
	x	y	z	x	y	z
**3D Systems**						
3D - $MAPbI_3$ (p-cub. met-off)	0.36	0.43	0.61	0.39	0.52	0.38
3D - $MAPbI_3$ (p-cub. met-on)	0.20	0.24	0.28	0.22	0.25	0.22
3D - $MAPbI_3$ (tet. met-off)	0.33	0.33	0.39	0.41	0.41	0.27
3D - $MAPbI_3$ (tet. met-on)	0.25	0.25	0.34	0.26	0.26	0.23
3D - $MAPbI_3$ (ort.)	0.38	0.29	0.35	0.32	0.25	0.29
3D - $MASnI_3$ (p-cub. met-off)	0.22	0.21	0.25	0.68	0.58	0.47
3D - $MASnI_3$ (p-cub. met-on)	0.17	0.19	0.20	0.39	0.43	0.28
3D - $MASnI_3$ (tet. met-off)	0.21	0.21	0.25	0.65	0.65	0.28
3D - $MASnI_3$ (tet. met-on)	0.20	0.20	0.24	0.47	0.47	0.24
3D - $MASnI_3$ (ort.)	0.25	0.22	0.22	0.36	0.32	0.34
**Quasi-2D Systems**						
2D - BA_2PbI_4 (met-off)	0.49	0.45	29.18	0.49	0.56	39.57
2D - BA_2PbI_4 (met-on)	0.22	0.19	18.42	0.20	0.19	19.52
2D - BA_2SnI_4 (met-off)	0.41	0.40	27.52	0.38	0.43	16.90
2D - BA_2SnI_4 (met-on)	0.20	0.17	33.02	0.18	0.19	16.48
**Quasi-1D Systems**						
1D - BA_3PbI_5 (met-off)	1.28	9.54	11.62	0.29	10.18	9.83
1D - BA_3PbI_5 (met-on)	5.90	33.30	70.27	0.27	68.15	14.76
1D - BA_3SnI_5 (met-off)	0.84	10.60	16.06	0.20	14.85	20.05
1D - BA_3SnI_5 (met-on)	4.29	28.96	19.56	0.19	12.35	16.56

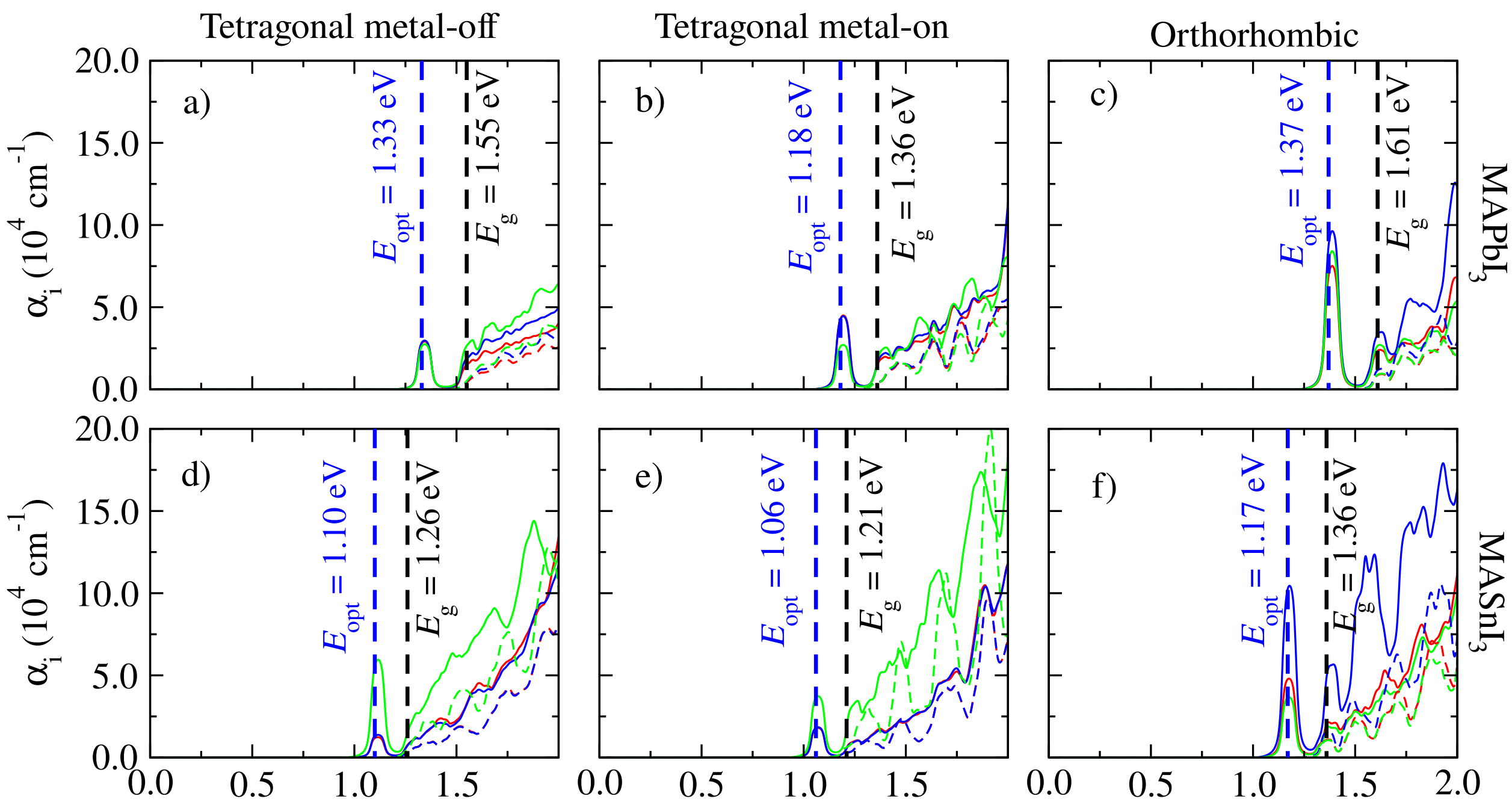

Figure S6. Absorption coefficient (α) calculated through independent particle approximation ((W)TB+IPA as dashed lines) and through (W)TB+BSE formalism (solid lines) for the excitonic effects. The red, blue, and green colors represent α on the x, y, and z axes. The values of electronic band gap (E_g) and optical band gap (E_{opt}) are depicted in each graphic. The panels refer to a) $MAPbI_3$ tetragonal metal-off, b) $MAPbI_3$ tetragonal metal-on, c) $MAPbI_3$ orthorhombic, d) $MASnI_3$ tetragonal metal-off, e) $MASnI_3$ tetragonal metal-on and f) $MASnI_3$ orthorhombic.

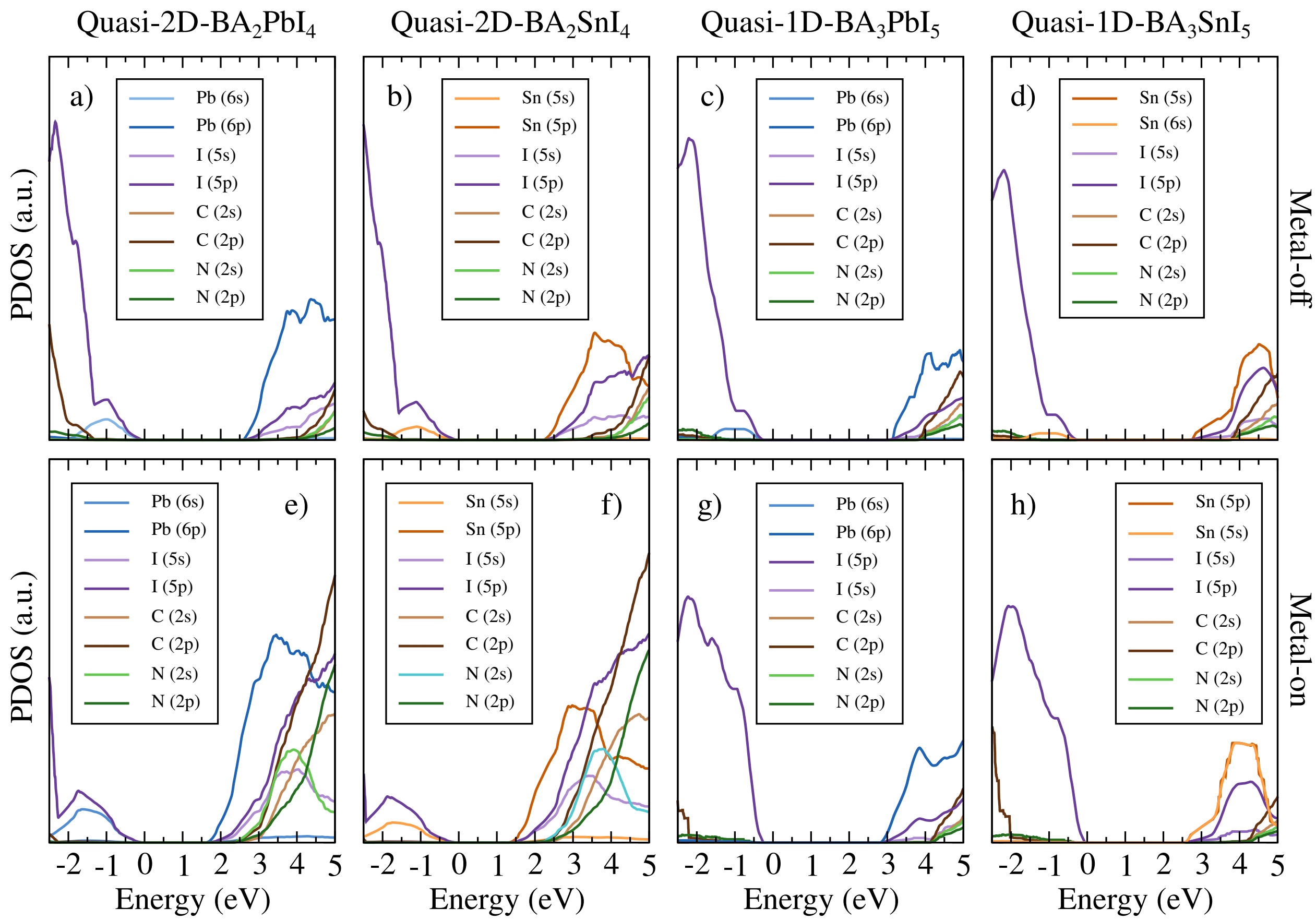

Figure S7. Projected density of states, in arbitrary units (a.u.), for our quasi-2D and quasi-1D systems regarding orbitals s and p contributions for the Pb (Sn), I, C and N. The first line depicts the PDOS obtained for metal-off systems, while the second line depicts the ones obtained for metal-on systems. The conduction band (CB) contributions were multiplied by a factor of 5. The panels refer to a) BA_2PbI_4 metal-off, b) BA_2SnI_4 metal-off, c) BA_3PbI_5 metal-off, d) BA_3SnI_5 metal-off, e) BA_2PbI_4 metal-on, f) BA_2SnI_4 metal-on, g) BA_3PbI_5 metal-on and h) BA_3SnI_5 metal-on.

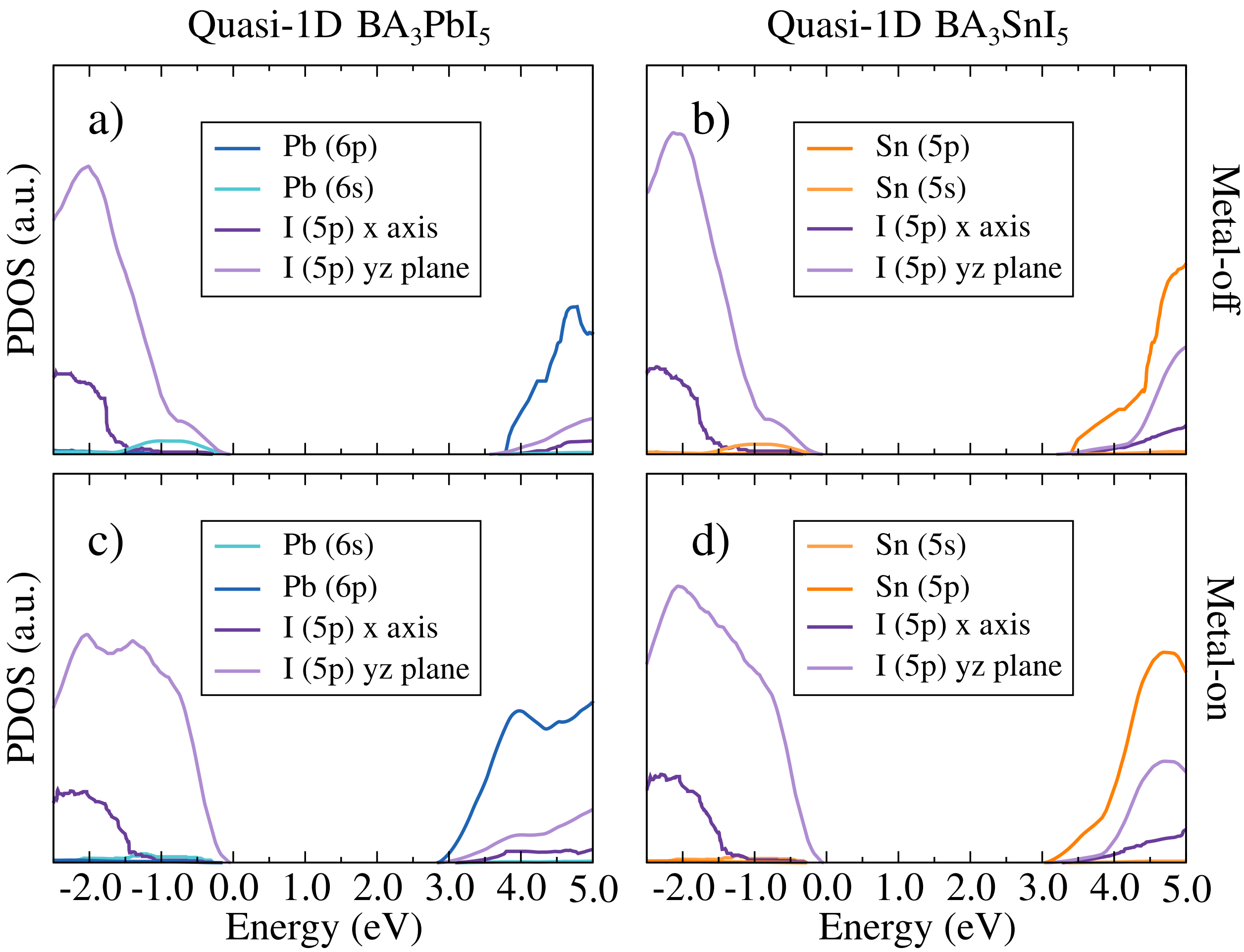

Figure S8. Local and projected density of states considering the 6s and 6p orbitals for Pb, and 5p orbitals for I. The iodine orbitals were separated along the x-axis (periodic direction) and in the yz plane (non-periodic direction). The conduction bands were multiplied by a factor of 7. The panels refer to a) Quasi-1D BA_3PbI_5 metal-off, b) Quasi-1D BA_3SnI_5 metal-off, c) Quasi-1D BA_3PbI_5 metal-on, d) Quasi-1D BA_3SnI_5 metal-on.

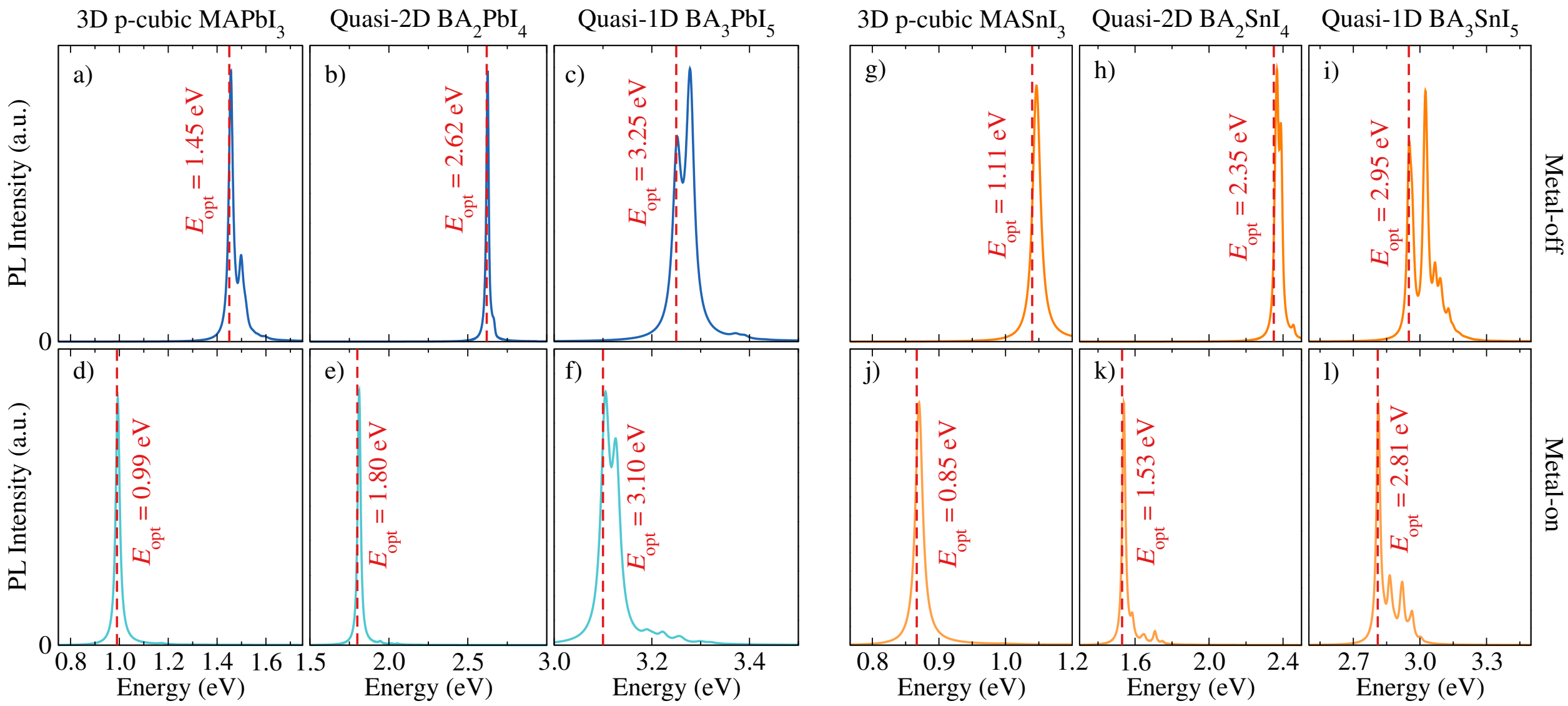

Figure S9. Calculated photoluminescence peaks, in arbitrary units, for representative systems of our investigation. The blue and cyan lines refer to the Pb-based systems, while the orange and light orange lines depict the Sn-based systems. The panels refer to a) 3D $MAPbI_3$ metal-off, b) quasi-2D BA_2PbI_4 metal-off, c) quasi-1D BA_3PbI_5 metal-off, d) 3D $MAPbI_3$ metal-on, e) quasi-2D BA_2PbI_4 metal-on, f) quasi-1D BA_3PbI_5 metal-on, g) 3D $MASnI_3$ metal-off, h) quasi-2D BA_2SnI_4 metal-off, i) quasi-1D BA_3SnI_5 metal-off, j) 3D $MASnI_3$ metal-on, k) quasi-2D BA_2SnI_4 metal-on, l) quasi-1D BA_3SnI_5 metal-on.

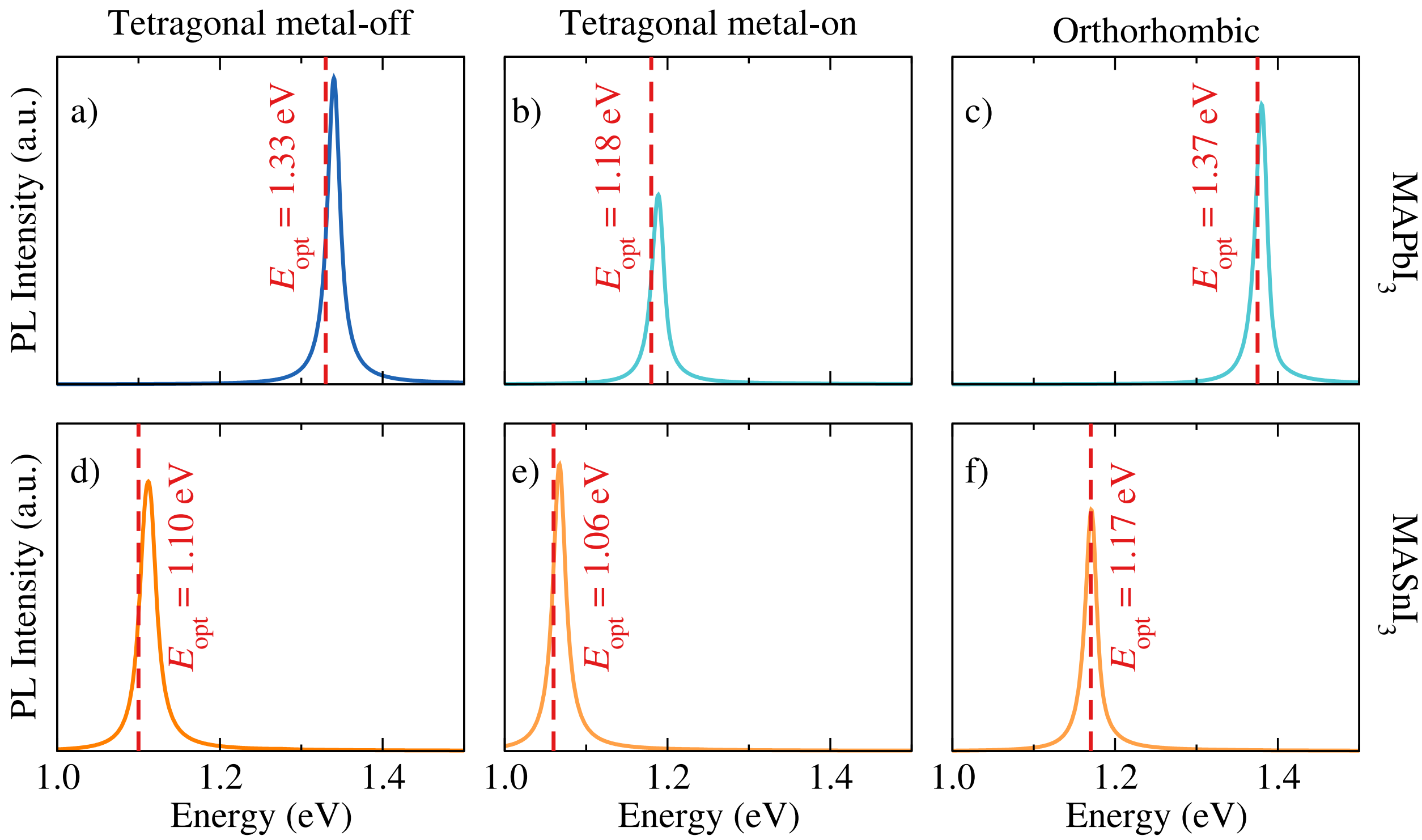

Figure S10. Calculated photoluminescence peaks, in arbitrary units (a.u.), for our 3D systems considering tetragonal and orthorhombic phases. The blue and cyan lines refer to the $MAPbI_3$ structures, whilst the orange and light orange lines refer to the $MASnI_3$ structures. The panels represent: a) $MAPbI_3$ tetragonal metal-off, b) $MAPbI_3$ tetragonal metal-on, c) $MAPbI_3$ orthorhombic, d) $MASnI_3$ tetragonal metal-off, e) $MASnI_3$ tetragonal metal-on, f) $MASnI_3$ orthorhombic.

References

(1) Bonadio, A.; Sabino, F. P.; Freitas, A. L.; Felez, M. R.; Dalpian, G. M.; Souza, J. A. Comparing the cubic and tetragonal phases of mapbi3 at room temperature. *Inorg. Chem.* **2023**, *62*, 7533–7544.

(2) Stoumpos, C. C.; Malliakas, C. D.; Kanatzidis, M. G. Semiconducting Tin and Lead Iodide Perovskites with Organic Cations: Phase Transitions, High Mobilities, and Near-Infrared Photoluminescent Properties. *Inorg. Chem.* **2013**, *52*, 9019–9038.

(3) Biega, R.-I.; Bokdam, M.; Herrmann, K.; Mohanraj, J.; Skrybeck, D.; Thelakkat, M.; Retsch, M.; Leppert, L. Dynamic Distortions of Quasi-2D Ruddlesden–Popper Perovskites at Elevated Temperatures: Influence on Thermal and Electronic Properties. *JPhysChemC* **2023**, *127*, 9183–9195.

(4) Li, Y.; Zhou, H.; Xia, M.; Shen, H.; Wang, T.; Gao, H.; Sheng, X.; Han, Y.; Chen, Z.; Dou, L.; others Phase-pure 2D tin halide perovskite thin flakes for stable lasing. *Sci. Adv.* **2023**, *9*, eadh0517.

(5) Jiang, L.; Wu, T.; Sun, L.; Li, Y.-J.; Li, A.; Lu, R.; Zou, K.; Deng, W.-Q. First-Principles Screening of Lead-Free Methylammonium Metal Iodine Perovskites for Photovoltaic Application. *J. Phys. Chem. C* **2017**, *121*, 24359–24364.

(6) Semenova, O.; Yudanova, E.; Yeryukov, N.; Zhivodkov, Y.; Shamirzaev, T.; Maximovskiy, E.; Gromilov, S.; Troitskaia, I. Perovskite $CH_3NH_3PbI_3$ crystals and films. Synthesis and characterization. *J. Cryst. Growth* **2017**, *462*, 45–49.

(7) Singh, S.; Li, C.; Panzer, F.; Narasimhan, K. L.; Graeser, A.; Gujar, T. P.; Köhler, A.; Thelakkat, M.; Huettner, S.; Kabra, D. Effect of Thermal and Structural Disorder on the Electronic Structure of Hybrid Perovskite Semiconductor $CH_3NH_3PbI_3$. *J. Phys. Chem. Lett.* **2016**, *7*, 3014–3021.

(8) Huang, Y.; Su, J.; Li, Q.; Wang, D.; Xu, L.; Bai, Y. Structure, optical and electrical properties of $CH_3NH_3SnI_3$ single crystal. *Physica B Condens. Matter* **2019**, *563*, 107–112.

(9) Gélvez-Rueda, M. C.; Peeters, S.; Wang, P.-C.; Felter, K. M.; Grozema, F. C. Effect of

structural defects and impurities on the excited state dynamics of 2D BA_2PbI_4 perovskite. *Helv. Chim. Acta* **2020**, *103*, e2000121.

(10) Hansen, K. R.; McClure, C. E.; Powell, D.; Hsieh, H.-C.; Flannery, L.; Garden, K.; Miller, E. J.; King, D. J.; Sainio, S.; Nordlund, D.; others Low exciton binding energies and localized exciton–polaron states in 2D tin halide perovskites. *Adv. Opt. Mater.* **2022**, *10*, 2102698.
